# Molecularly Engineered Supramolecular Thermoresponsive Hydrogels with Tunable Mechanical and Dynamic Properties

**DOI:** 10.1021/acs.biomac.3c01357

**Published:** 2024-07-26

**Authors:** Laura Rijns, Heleen Duijs, René P. M. Lafleur, Ruth Cardinaels, Anja R. A. Palmans, Patricia Y. W. Dankers, Lu Su

**Affiliations:** † Department of Biomedical Engineering, Institute for Complex Molecular Systems (ICMS), Eindhoven University of Technology, Eindhoven 5600 MB, The Netherlands; ‡ Leiden Academic Centre for Drug Research (LACDR), Leiden University, Wassenaarseweg 76, Leiden 2333 AL, The Netherlands; § Laboratory of Macromolecular and Organic Chemistry, Institute for Complex Molecular Systems (ICMS), Department of Chemical Engineering and Chemistry, Eindhoven University of Technology, Eindhoven 5600 MB, The Netherlands; ∥ Processing and Performance of Materials, Institute for Complex Molecular Systems (ICMS), Department of Mechanical Engineering, Eindhoven University of Technology, Eindhoven 5600 MB, The Netherlands; ⊥ Soft Matter, Rheology and Technology, Department of Chemical Engineering, KU Leuven, Leuven 3001, Belgium

## Abstract

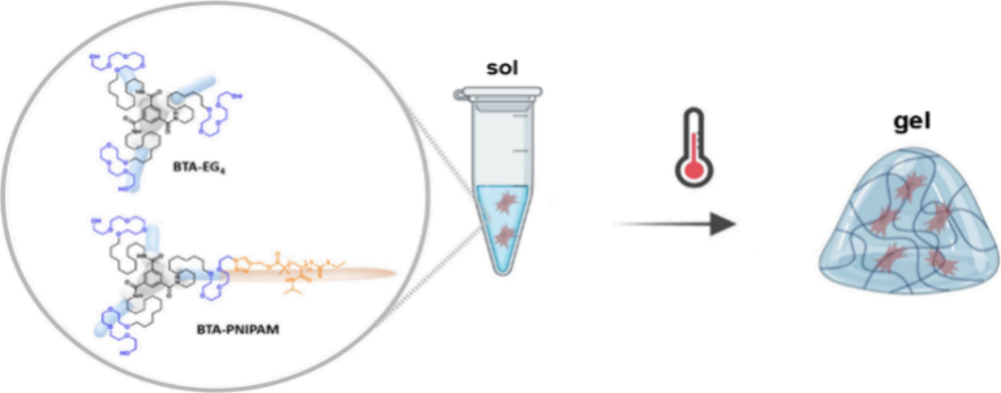

Synthetic supramolecular polymers and hydrogels in water are emerging as promising biomaterials due to their modularity and intrinsic dynamics. Here, we introduce temperature sensitivity into the nonfunctionalized benzene-1,3,5-tricarboxamide (**BTA-EG**_**4**_) supramolecular system by incorporating a poly(*N*-isopropylacrylamide)-functionalized (**BTA-PNIPAM)** moiety, enabling 3D cell encapsulation applications. The viscous and structural properties in the solution state as well as the mechanical and dynamic features in the gel state of **BTA-PNIPAM/BTA-EG**_**4**_ mixtures were investigated and modulated. In the dilute state (*c* ∼μM), **BTA-PNIPAM** acted as a chain capper below the cloud point temperature (*T*_cp_ = 24 °C) but served as a cross-linker above *T*_cp_. At higher concentrations (*c* ∼mM), weak or stiff hydrogels were obtained, depending on the **BTA-PNIPAM/BTA-EG_4_** ratio. The mixture with the highest **BTA-PNIPAM** ratio was ∼100 times stiffer and ∼10 times less dynamic than **BTA-EG**_**4**_ hydrogel. Facile cell encapsulation in 3D was realized by leveraging the temperature-sensitive sol–gel transition, opening opportunities for utilizing this hydrogel as an extracellular matrix mimic.

## Introduction

The extracellular matrix (ECM) is a dynamic, multicomponent network, consisting of a mixture of glycosaminoglycans, proteins, and biomolecules, that surrounds cells.^1^ The ECM provides not only structural and physical support to cells, but its mechanical and dynamic properties are important determinants in regulating cellular behavior.^2−5^ To illustrate, matrix elasticity determines the specification of stem cell lineage.^2^ Additionally, remodeling of the ECM is crucial for healthy morphogenesis of most organs, such as intestines and lungs.^6−9^ This ECM degradation is mostly regulated through enzymes, like metalloproteases.^10^ Importantly, any discrepancy in ECM stiffness and dynamics might contribute to the development of diseases, like fibrosis^11−14^ or cancer.^15−19^ With the goal to better understand the influence of the mechanical and dynamic properties of the ECM on cellular behavior, many different material types have been developed as mimics of the ECM.^20−27^

**Figure 1:**
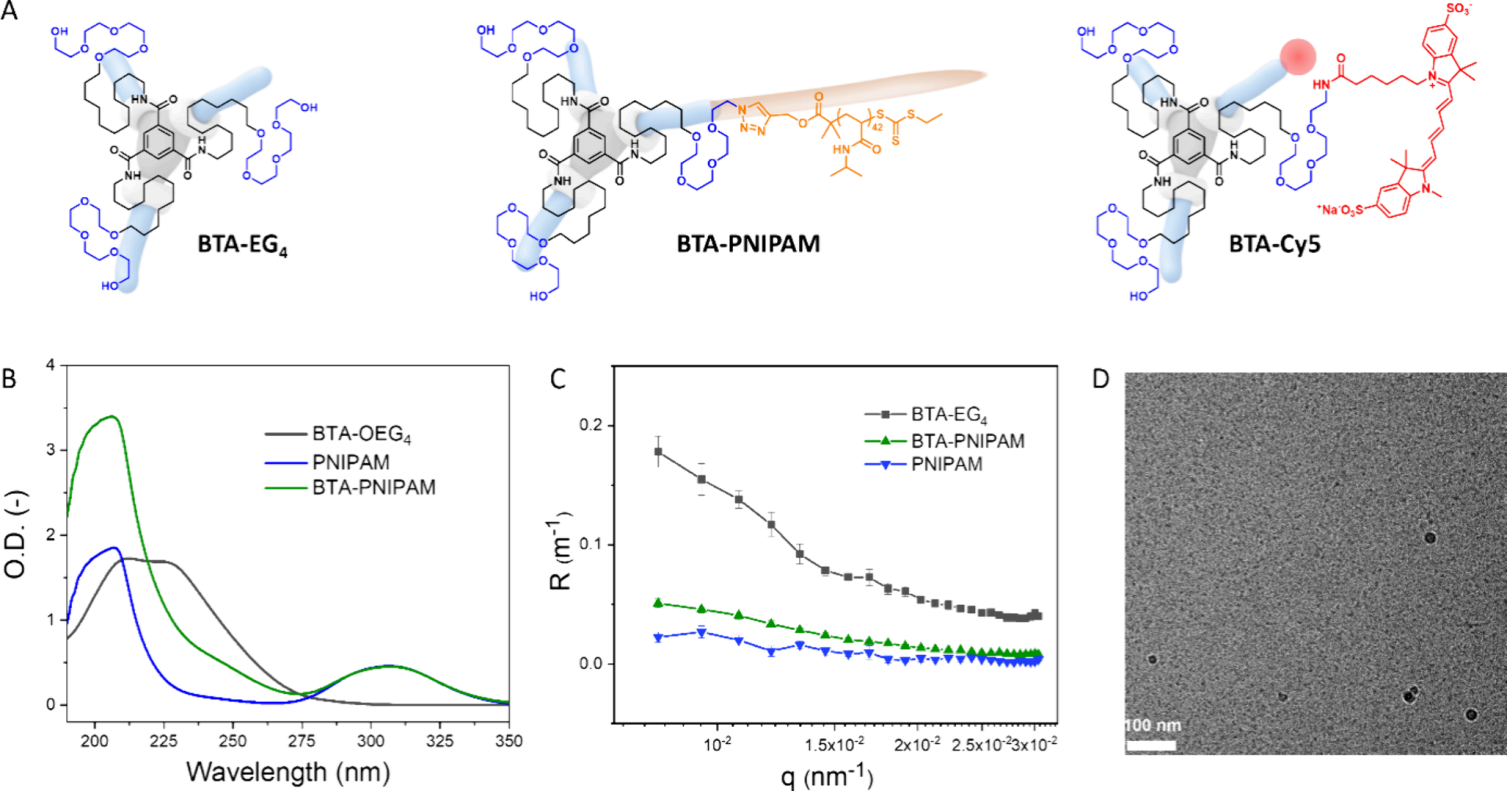
Molecular design and characterization in the dilute state of homoassemblies. (A) Chemical structures of the supramolecular building blocks, **BTA-EG**_**4**_, **BTA-PNIPAM**, and **BTA-Cy5**. (B) UV–vis spectra (50 μM) and (C) SLS profiles (500 μM) of **BTA-EG**_**4**_, **BTA-PNIPAM**, and PNIPAM in MQ-H_2_O at 10 °C. (D) Cryo-TEM image of **BTA-PNIPAM** at the concentration of 4.8 mM in MQ-H_2_O at 10 °C, showing spherical micellar structures. Scale bar: 100 nm. The large black spherical particles are ice-crystals.

Herein, supramolecular polymers and hydrogels are emerging as an attractive class of ECM mimics owing to their high modularity and intrinsic dynamics.^28−30^ Supramolecular polymers are formed through noncovalent interactions between their monomers, such as hydrogen bonds, hydrophobic interactions, and π–π stacking. We have ample experience with the benzene-1,3,5-tricarboxamide (BTA) moiety as the supramolecular building block.^31,32^ When the BTA core is equipped three times with C_12_ hydrophobic spacers followed by a tetra(ethylene glycol) for water solubility, it forms into micrometer-long double helical fibers through a combination of triple amide hydrogen bonding, π–π stacking, and the hydrophobic effect.^33^ We refer to this BTA as nonfunctionalized BTA (**BTA-EG**_**4**_, Figure 1A) in this paper. Supramolecular hydrogels based on (carbohydrate-functionalized) BTAs were successfully engineered by increasing the total BTA concentration or through the addition of a bifunctional BTA cross-linker molecule.^34−38^ More recently, the crucial interplay between the mechanical and dynamic properties of BTA supramolecular hydrogels was revealed in dictating cell spreading behavior in 2D:^37^ hydrogel dynamics dictate cellular spreading on soft hydrogels, but is overruled by stiffness on stiffer hydrogels. Additionally, we introduced responsiveness into the BTA supramolecular system, realizing dilution-induced, reversible gel–sol transitions.^32^

To further explore and expand the responsiveness of the BTA system, we here engineer thermoresponsiveness into BTA supramolecular hydrogels by introducing a temperature-responsive poly(*N*-isopropylacrylamide)-functionalized supramolecular monomer **BTA-PNIPAM** (Figure 1A). PNIPAM is hydrophilic at low temperatures, but when heated above its lower critical solution temperature (LCST), it undergoes a hydrophobic collapse.^39,40^ Others successfully used PNIPAM-functionalized hydrogels to create stimuli-responsive hydrogels, for fundamental understanding,^41^ but also for distinct applications, such as electronic devices,^42−44^ mimicking cytoskeletal stiffening,^45^ 3D printing,^46−48^ wound-healing dressings,^49−51^ and cell culture.^52−54^

In this study, a well-defined PNIPAM polymer chain was covalently attached onto the BTA core, to create a thermoresponsive supramolecular building block **BTA-PNIPAM** (Figure 1A). The solution and hydrogel properties were modulated by tuning the ratio between **BTA-PNIPAM** and **BTA-EG**_**4**_. The properties and thermoresponsiveness in the dilute state of both homo- and coassemblies were studied using a combination of UV spectroscopy, light scattering, cryogenic transmission electron microscopy (cryo-TEM), and viscosity measurements. The thermoresponsiveness of the hydrogel and its mechanical and dynamic properties were investigated using rheology and fluorescence recovery after photobleaching (FRAP), respectively. Furthermore, facile cell encapsulation in 3D is realized by utilizing the temperature-sensitive sol–gel transition, demonstrating the potential of **BTA-PNIPAM/BTA-EG**_**4**_ hydrogels as extracellular matrix mimics.

## Experimental Section

### Materials

All chemicals were purchased from Sigma-Aldrich and used as received without further purification unless otherwise noted. Compounds *N*^1^-(1-azido-3,6,9,12-tetraoxatetracosan-24-yl)-*N*^3^,*N*^5^-bis(1-hydroxy-3,6,9,12-tetraoxatetracosan-24-yl)benzene-1,3,5-tricarboxamide (**BTA-mN**_**3**_),^55^*N*^1^,*N*^3^,*N*^5^-tris(dodecyl-tetra(ethylene glycol)benzene-1,3,5-tricarboxamide (**BTA-EG**_**4**_),^31^ fluorescent-labeled **BTA-Cy5**,^55^ cyclic RGD-modified BTA (**BTA-cRGD**, Figure S17),^37^ and *S*-ethyl-S′-(α,α′-dimethyl-α″-acetic acid)trithiocarbonate (EMP)^56^ were synthesized according to literature procedures. *N*-Isopropylacrylamide (NIPAM) was recrystallized three times from benzene/hexane (65:35 v/v) prior to use. Azobis(isobutyronitrile) (AIBN) was recrystallized twice from methanol prior to use. Water was purified on an EMD Milipore Mili-Q integral water purification system. Dry solvents were obtained with an MBRAUN solvent purification system (MB-SPS). Reactions were followed by thin-layer chromatography (precoated 0.25 mm, 60-F254 silica gel plates from Merck). Automated column chromatography was performed using Biotage SNAP-KP SIL cartridges. Normal-phase column chromatography was performed on a CombiFlash Rf4x instrument (Teledyne ISCO) with RediSep Rf columns. Reversed-phase column chromatography was performed on a Biotage with SNAP-KP xC-18-SH cartridges.

### Instrumentation and Methods

#### Nuclear Magnetic Resonance (NMR)

NMR spectra were recorded on Bruker 400 MHz Ultrashield spectrometers (400 MHz for ^1^H NMR). Deuterated solvents used are indicated in each case. Chemical shifts (δ) are expressed in parts per million and refer to the residual peak of the solvent. Peak multiplicity is abbreviated as s: singlet; d: doublet; t: triplet; b: broad; q: quartet; qd: quartet of doublets; m: multiplet.

#### Matrix-Assisted Laser Absorption/Ionization-Time-of-Flight (MALDI-TOF) Mass Spectra

MALDI-TOF was obtained on a PerSeptive Biosystems Voyager DE-PRO spectrometer using α-cyano-4-hydroxycinnamic acid (CHCA) or trans-2-[3-(4-*tert*-butylphenyl)-2-methyl-2-propenylidene]-malononitrile (DCTB) as a matrix.

#### Fourier Transform Infrared (FT-IR) Spectra

FT-IR spectra were recorded by using a PerkinElmer Spectrum Two FT-IR spectrometer equipped with a PerkinElmer Universal ATR Two Accessory.

#### Size-Exclusion Chromatography (SEC)

SEC was used to determine the polymer molar mass and molar mass distribution (or dispersity, *Đ*). Polymer solutions were prepared at a known concentration (2 mg/mL) in *N,N*-dimethylformamide (DMF), and 200 μL of injection volume was used. After filtration through a 0.45 μm PTFE filter, the polymer samples were passed through the SEC system equilibrated at 40 °C in DMF as the mobile phase with a flow rate of 1 mL/min. SEC was carried out in PL-GPC-50 plus from Polymer Laboratories (Agilent Technologies) with the refractive index detector working in DMF containing 10 mM LiBr at 50 °C on a Shodex GPC-KD-804 column (exclusion limit = 400 kDa; 0.8 cmi.d. ×300 mL), which was calibrated with poly(ethylene oxide) samples with a range from 282 to 77,350 Da (Polymer Laboratories-Agilent Technologies).

#### Ultraviolet–Visible (UV–vis) Absorbance Spectra

UV–vis spectra were recorded on a Jasco V-650 UV–vis spectrometer equipped with a Jasco ETCT-762 temperature controller. Measurements were performed using Quartz cuvettes with a path length of 10 mm. All spectra were averaged over three measurements.

#### Cryo-TEM

Vitrified films were prepared in a “Vitrobot” instrument (FEI VitrobotTM Mark III, FEI Company) at predetermined temperature and at a relative humidity of 100%. In the preparation chamber of the “Vitrobot”, a 3 μL sample was applied on a Lacey film (LC200-CU, Electron Microscopy Sciences). These films were surface plasma treated just prior to use (Cressington 208 carbon coater operating at 5 mA for 40 s). An excess sample was removed by blotting using filter paper for 3 s at −3 mm, and the thin film thus formed was shot (acceleration about 3*g*) into liquid ethane just above its freezing point. Vitrified films were transferred into the vacuum of a CryoTITAN equipped with a field emission gun that was operated at 300 kV, a postcolumn Gatan energy filter, and a 2048 × 2048 Gatan CCD camera. Microscopy images were taken at low-dose conditions for the vitrified samples, starting at a magnification of 6500 nm with a defocus setting of 40 μm. Subsequently, images were acquired at 24,000 magnification and at a defocus of 10 or 15 μm. High-magnification TEM images were acquired at a magnification of 24,000 and at a defocus of 10 μm using normal-dose conditions.

#### Dynamic Light Scattering (DLS)

DLS measurements were performed on an ALV Compact Goniometer System (CGS-3) instrument equipped with an ALV-7004 Digital Multiple Tau Real Time Correlator and 532 nm solid state laser (40 mW). Scattering data were recorded at 90° and 135°, respectively, in five runs for each sample condition. Samples were filtered using Waterman syringe filters with pores of 0.2 μm and held in disposable tubes of glass with an outer diameter of 10 mm. Afterward, the ALV software (Dullware Inc.) based on the CONTIN algorithm was used to obtain the apparent hydrodynamic radius (*R*_h_). No obvious scattering angle dependence of *R*_h_ was observed for **BTA-PNIPAM**.

#### Static Light Scattering (SLS)

Measurements were performed on an ALV Compact Goniometer (CGS-3) Multi-Detector (MD-4) equipped with an ALV-7004 Digital Multiple Tau Real Time Correlator and Nd:YAG laser operating at a wavelength (λ) of 532 nm. Scattering intensities (*I*) were recorded at detection angles (θ) ranging from 30° to 150° in increments of 5°, in 10 runs of 10 s per angle. Scattering intensities were corrected for the sample holder, solvent, and scattering volume according to
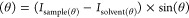
1where *I*_sample_ and *I*_solvent_ are scattering intensities measured for the sample and solvent, respectively. Detection angles were converted to scattering vectors (*q*) according to
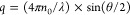
2where *n*_0_ = 1.332 is the refractive index of water.

#### Rheology

Rheological measurements were performed on a TA Instruments DHR-3 rheometer (TA Instruments) or an Anton Paar rheometer (MCR-501). For measurements on the DHR-3 rheometer, a solution/hydrogel was deposited onto the rheometer stage and incubated for 10 s before all of the measurements. A 20 mm aluminum cone with a 2° cone angle was used, and the gap height was set to the truncation height of 56 μm. For measurements on the MCR-501 rheometer, a cone plate (d = 25 mm) with a gap height of 49 μm was used. The temperature was controlled using a Peltier system, and a water trap prevented sample evaporation. Each measurement was repeated multiple times with different batches to ensure data reliability. The storage modulus *G*′ and loss modulus *G*″ were monitored under an applied strain of 0.1–1000% at a frequency of 1 rad s^–1^ for the strain sweep, and a frequency of 100–0.1 rad s^–1^ at strain of 1% for the frequency sweep, the latter being in the linear region. Temperature sweeps were performed from 10 to 40 °C with a heating/cooling rate of 1 °C/min. Temperature-reversible experiments were carried out by subjecting the gel to a strain of 1% and frequency of 1 rad s^–1^. A time sweep experiment of **G-1** was measured at 1% strain and 1 rad/s at 37 °C.

#### Viscosity

Viscosity measurements were also performed on a TA Instruments DHR-3 rheometer (TA Instruments) using similar settings to those mentioned above in Rheology. To determine the viscosity, the flow peak hold mode at a shear rate of 1 s^–1^ was applied with a duration of 60 s. For the viscosity tests, a solution was deposited onto the rheometer stage and incubated for 10 s at 10 or 25 °C before the measurements.

#### Fluorescence Recovery after Photobleaching (FRAP)

FRAP measurements were carried out using a Leica TCS SP5 inverted confocal microscope (Leica Microsystems) equipped with a 20× objective (HCX PL APO CS 20.0 × 0.70 DRY UV) or a 40× objective (HCX PL APO CS 40.0 × 1.1 water UV). The samples, each containing 20 μM **BTA-Cy5**, were prepared according to the procedure described below in Sample Preparation and stored in a glass vial at 4 °C. Prior to measuring, the samples were kept on ice, vortexed, and pipetted into an Ibidi microangiogenesis glass-bottom well plate. To minimize sample drying during the measurements, the empty wells were filled with water. Exchange dynamics experiments were carried out via sample illumination using white laser at λ = 633 nm for Cy5 excitation. Emission was collected at λ = 645–735 nm using a hybrid detector. A circular area with a diameter of 20 or 50 μm was photobleached at 100% laser power for 50 or 100 frames (0.653 frames/s), and the postbleaching time-lapse imaging was performed for maximum 6 h, depending on the sample. To correct for drift of the hydrogel, the region of interest (ROI) was adjusted manually. Image analysis was performed in Leica AF software and data processing in Origin. The fluorescence half-life time (τ_1/2_), diffusion constant (*D*), time until plateau fluorescence intensity was reached, and immobile fractions were evaluated during data analysis. Data were normalized by dividing the fluorescence intensity by the maximum observed fluorescence intensity in a nonbleached circular area of similar size for each measurement point. The fluorescence recovery data points were fitted using a single exponential growth model. *D* was calculated assuming a pure isotropic diffusion model based on Soumpasis’ work^57^ using eq 3:
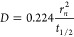
3in which *r_n_* is the radius of the uniform bleach laser in μm, τ_1/2_ is the fluorescence half-life time in s, and the coefficient 0.224 is numerically determined.^58^

#### Cell Culture

Human normal dermal fibroblasts (hNDFs) were cultured in a DMEM-advanced medium (Gibco) supplemented with 10 v/v% fetal bovine serum (FBS), 1 v/v% penicillin–streptomycin (P/S), and 1 v/v% Gluta MAX at 37 °C and 5% CO_2_. The medium was changed every 5 days, and cells were passaged using trypsin/EDTA at 90% confluency. Cells were cultured from the 11th passage until 14th passage. Cells were encapsulated inside supramolecular hydrogels as stated in the Sample Preparation section (BTA-EG4/BTA-PNIPAM Solution and Hydrogel Preparation for Cell Encapsulation). Cells were cultured inside hydrogels for 1 day.

#### Cell Viability Assay

An MTT (3-[4,5-dimethylthiazol-2-yl]-2,5-diphenyltetrazolium bromide) assay was executed to evaluate the cell viability of hNDFs encapsulated inside the **G-0, G-0.1, G-0.5**, and **G-1** hydrogels. Samples were prepared in a 96-well U-bottom plate (Greiner Bio-One GmbH, Alphen aan den Rijn, The Netherlands) on ice, by mixing 40 μL of **G-0, G-0.1, G-0.5**, or **G-1** with 20 μL of cells (10,000 cells per gel, per well) in culture medium, to ensure the cells were homogeneous embedded into the *in-situ* formed hydrogel. After 30 min of incubation at 37 °C with 5% CO_2_, 100 μL of culture medium was carefully added in a dropwise manner to each well. After another 24 h incubation, the medium was removed, and 200 μL of MTT (0.5 mg/mL in Opti-MEM) was added to each well. The plate was placed in the incubator at 37 °C with 5% CO_2_ for 3 h. Viable cells convert MTT into formazan crystals. Cells without hydrogel were used as a positive control and cells treated with 70% ethanol were used as a negative control. After 3 h incubation, 150 μL of medium was removed and supplemented with 100 μL of DMSO to solubilize the formazan crystals (total volume 150 μL now). After 20 min of gentle shaking, absorbance was measured at 590 nm, and at 690 nm as a reference, using a Spark Microplate Reader (Tecan Austria GmbH). The reference absorbance value was subtracted from the absorbance values of all samples. Cell viability was then normalized against the cells that were cultured without hydrogel, which was set at 100% viability. Data are plotted as mean with S.D., with *n* = 6 per condition.

#### Immunofluorescence Staining and Confocal Fluorescence Microscopy

Cells encapsulated inside hydrogels were cultured for 1 day at 37 °C with 5% CO_2_. Next, the samples were washed with PBS, followed by fixation for 10 min with 3.7 v/v% formaldehyde in PBS, and washed with PBS twice afterward. Afterward, samples were stained with phalloidin in 0.2 v/v% Triton X-100 in PBS (1:200) to stain F-actin for 30 min at 37 °C in the dark to visualize the cellular cytoskeleton. Finally, the samples were washed with PBS containing 0.2 v/v% Triton X-100. Immediately thereafter, the samples were imaged (and only mounted if necessary) on a Leica TCS SP5 inverted confocal microscope using 20× (HCX PL APO CS 20.0 × 0.7 DRY UV) and 40× (HCX PL APO CS 40.0 × 1.1 water UV) objectives. Image analysis and quantification of circularity (*n* is at least 10 cells per condition) was performed using LAS X and Fiji (ImageJ) software. Quantified data are represented as mean with standard error of the mean (SEM).

#### Live/Dead Imaging

Live/dead cell staining was performed to monitor cell viability as well as cell distribution through the hydrogels. Hydrogel **G-1** (120 μL) with **BTA-cRGD** was prepared by mixing 4,000 cells in 40 μL of culture medium with 80 μL of **G-1** on ice, following the method as stated in the Sample Preparation section (BTA-EG4/BTA-PNIPAM Solution and Hydrogel Preparation for Cell Encapsulation), in a 96-well Screenstar plate for microscopy (Greiner Bio-One GmbH, Alphen aan den Rijn, The Netherlands). The cells were incubated for 10 min at 37 °C with 5% CO_2_, after which 100 μL of culture medium was added to each well. After 24 h incubation, 120 μL of medium was removed and 100 μL of dye solution was added, according to the protocol of the LIVE/DEAD Cell Imaging Kit (488/570) (Thermo Fischer, catalog number R37601). The cells were incubated for 15 min at room temperature and imaged on a Nikon Eclipse Ti inverted confocal microscope using 20× objective (Nikon Plan Apo 20×/0.75 DIC N2). A 3D image was constructed with 21 slices along the *z*-axis recorded every 5 μm. Cells without hydrogel were used as a positive control and cells treated with 70% ethanol as a negative control, with *n* = 3 for each condition. Analysis of the images and construction of 3D movies were performed with Fiji (ImageJ) software.

### Synthetic Procedures

#### Synthesis of Prop-2-yn-1-yl 2-(((ethylthio)carbonothioyl)thio)-2-methylpropanoate (CTA)

To a solution of *S*-ethyl-S′-(α,α′-dimethyl-α″-acetic acid)trithiocarbonate (8.0 g, 35.7 mmol) and propargyl alcohol (4.0 g, 71.4 mmol) in 100 mL of dichloromethane (DCM), *N*,*N*′-diisopropylcarbodiimide (DIC, 9.0 g, 71.4 mmol) was added dropwise. The reaction mixture was stirred overnight at room temperature, filtered, and concentrated under reduced pressure. The crude product was purified via column chromatography (*n*-hexane:ethyl acetate = 10:1), yielding a yellow oil (7.3 g, 78%). ^1^H NMR (400 MHz, CDCl_3_) δ ppm 4.70 (d, *J* = 2.5 Hz, 2H), 3.29 (q, *J* = 7.4 Hz, 2H), 2.47 (t, *J* = 2.5 Hz, 1H), 1.71 (s, 6H), 1.33 (t, *J* = 7.4 Hz, 3H). ^13^C NMR (100 MHz, CDCl_3_) δ ppm, 220.82, 172.32, 77.31, 75.10, 55.60, 53.31, 31.23, 25.21, 12.88. FT-IR (ATR, cm^–1^) 3702–3120, 2971, 1584, 1539, 1455, 1367, 1169, 1129, 1080, 810.

#### RAFT Polymerization to Afford PNIPAM

To a 25 mL round-bottom flask equipped with a magnetic stirring bar dried in an oven were added CTA (139.1 mg, 0.53 mmol), NIPAM (3.0 g, 26.5 mmol), AIBN (4.3 mg, 0.026 mmol), and 8 mL of 1,4-dioxane. The mixture was deoxygenated through bubbling with Ar while stirring for 10 min at room temperature. The reaction mixture was then immersed into a preheated oil bath at 65 °C to start the polymerization. The polymerization was quenched after 2.5 h by cooling the reaction flask with liquid N_2_. The polymer was purified by precipitation into 200 mL of diethyl ether twice. The product was collected and dried *in vacuo* for 24 h to obtain the polymer as a yellow solid. *M*_n,SEC_ = 2.8 kDa, and *Đ* = 1.08. *M*_n,NMR_ = 5.0 kDa. ^1^H NMR (400 MHz, CDCl_3_) δ ppm 6.23 (s, 41H), 4.76–4.47 (b, 2H), 4.16–3.82 (m, 44H), 3.36 (qd, *J* = 7.5, 4.3 Hz, 2H), 2.78 (s, 15H), 2.40–1.48 (m, 113H), 1.46–1.27 (m, 22H), 1.25–0.84 (m, 276H), 8.39 (s, 3H), 7.85 (s, 1H), 7.17–5.91 (b, 48H), 5.18 (b, 2H), 4.56 (b, 2H), 3.99 (s, 45H), 3.88 (s, 2H), 3.72 (t, *J* = 4.6 Hz, 4H), 3.68–3.55 (m, 41H), 3.50–3.28 (m, 17H), 2.39–1.48 (m, 136H), 1.42–1.23 (m, 75H), 1.14 (s, 285H). ^13^C NMR (100 MHz, CDCl_3_) δ ppm, 221.02, 173.96, 91.65, 83.12, 42.56, 41.28, 34.58, 22.66, 12.88. FT-IR (ATR, cm^–1^) 3647–3159, 3072, 3027–2799, 1733, 1641, 1537, 1459, 1388, 1367, 1269, 1172, 1130, 924, 877, 839.

#### Azide–Alkyne Cycloaddition Reaction to Afford **BTA-PNIPAM**

An oven-dried flask containing a magnetic stirring bar was charged with **BTA-mN**_**3**_ (201.5 mg, 153.5 μmol), PNIPAM (500.6 mg, 99.72 μmol), *N,N,N′,N′,N″*-pentamethyldiethylenetriamine (PMDETA, 17.4 mg, 99.6 μmol), and 5 mL of dimethylacetamide (DMAc). The reaction mixture was deoxygenated through bubbling with argon, during which copper(I) bromide (6.6 mg, 45 μmol) was added. The flask was allowed to stir overnight under Ar. The solution mixture was passed through a reverse-phase column (MQ water and methanol as gradient eluents) to afford purified **BTA-PNIPAM** as a light yellowish powder after lyophilization (382.9 mg, 60.4% yield). *M*_n,SEC_ = 4.2 kDa, and *Đ* = 1.05. *M*_n,NMR_ = 5.0 kDa. ^1^H NMR (400 MHz, CDCl_3_) δ ppm 8.39 (s, 3H), 7.85 (s, 1H), 7.17–5.91 (b, 48H), 5.18 (b, 2H), 4.56 (b, 2H), 3.99 (s, 45H), 3.88 (s, 2H), 3.72 (t, *J* = 4.6 Hz, 4H), 3.68–3.55 (m, 41H), 3.50–3.28 (m, 17H), 2.39–1.48 (m, 136H), 1.42–1.23 (m, 75H), 1.14 (s, 285H). ^13^C NMR (100 MHz, CDCl_3_) δ ppm, 221.02, 174.18, 72.68, 71.56, 70.58, 70.54, 70.51, 70.24, 70.01, 61.62, 42.41, 41.31, 40.44, 29.51, 27.02, 26.03, 22.65, 14.10. FT-IR (ATR, cm^–1^) 3671–3157, 3075, 3003–2769, 1641, 1540, 1459, 1387, 1369, 1271, 1176, 1129, 926, 881, 837.

### Sample Preparation

The formulation procedure utilized to prepare supramolecular hydrogels is known to largely impact the hydrogel’s mechanical and dynamic properties.^59^ Therefore, fixed procedures were followed throughout this study to prepare different supramolecular solutions and hydrogels.

#### BTA-EG_4_ Solution or Hydrogel

The solid **BTA-EG**_**4**_ was weighed and mixed with MQ water to reach the desired concentration. The mixture was stirred at 80 °C for 15 min. The resulting hot and hazy mixture was vortexed for 15 s and allowed to equilibrate at room temperature (20 °C) overnight. Solution is referring to diluted state (i.e., below the critical gelation concentration, CGC = 9–10 mg/mL), while hydrogel is referring to a concentration above the CGC).

#### **BTA-PNIPAM** and PNIPAM Solution

**BTA-PNIPAM** or PNIPAM was directly dissolved in MQ water to afford a concentrated stock solution (50 or 100 mg/mL) and stored in a refrigerator (4 °C) for further use.

#### **BTA-EG_4_**/**BTA-PNIPAM** and **BTA-EG_4_**/PNIPAM Solution or Hydrogel

The **BTA-EG**_**4**_ solution or hydrogel was prepared according to the above-mentioned method. Cold **BTA-PNIPAM** or PNIPAM stock solution was mixed with **BTA-EG**_**4**_ solution or hydrogel and was allowed to equilibrate in an ice-bath to afford the hybrid **BTA-EG**_**4**_**/BTA-PNIPAM** or **BTA-EG**_**4**_**/**PNIPAM solution or hydrogel.

#### **BTA-EG_4_**/**BTA-PNIPAM** Solution and Hydrogel Preparation for Cell Encapsulation

The **BTA-EG**_**4**_ solution or hydrogel was prepared according to the above-mentioned method. Likewise, solid **BTA-cRGD** was weighed and mixed with MQ water to obtain the desired concentration. The mixture was stirred at 80 °C for 15 min. For sample **G-1** with 1 mM **BTA-cRGD** ([BTA]_total_ = 1 wt %, of which 0.83 wt % (8.3 mg/mL) **BTA-EG**_**4**_ and 0.17 wt % (1.7 mg/mL) **BTA-cRGD**), the **BTA-cRGD** and **BTA-EG**_**4**_ solutions were immediately pipetted together after heating as viscous liquids at the desired ratio. Cells (1 × 10^3^ cells per gel to ensure single cells) were mixed with **BTA-EG**_**4**_/**BTA-cRGD** solution in the desired ratio and amount. This low seeding density was utilized to ensure only cell–matrix interactions were investigated and cell–cell interactions were excluded. Then, **BTA-EG**_**4**_/**BTA-cRGD** solution with cells was pipetted into **BTA-PNIPAM** solution on ice. The mixture containing the cells was pipetted up and down several times to ensure homogeneous mixing. Thereafter, it was placed in the incubator at 37 °C to allow the formation of a gel, which took a few minutes depending on the solution volume (Figure S15). After 30 min incubation, the growth medium was added on top of the hydrogels in a very careful, dropwise manner (see cell culture section for more details). No cell sedimentation was observed. For cell culture experiments, 20 μL of cells was mixed with a total of 40 μL of hydrogel, yielding a total volume of 60 μL of hydrogel with cells encapsulated inside, unless stated otherwise.

## Results and Discussion

### Molecular Design and Synthesis

**BTA-PNIPAM** (Figure 1A) was synthesized via an azide–alkyne Huisgen cycloaddition between monoazide BTA (BTA-mN_3_) and alkyne functionalized PNIPAM with an overall yield of 55% (Scheme S1). The structure of **BTA-PNIPAM** was confirmed by ^1^H NMR and FT-IR spectroscopy (Figures S1 and S2). Size-exclusion chromatography (SEC) showed a clean shift to lower retention times as compared to the free PNIPAM precursor while maintaining a narrow distribution with *Đ* = 1.05 (Figure S3). Matrix-assisted laser desorption ionization time-of-flight mass spectrometry (MALDI-TOF-MS) displayed a right shift toward larger *m*/*z* value for **BTA-PNIPAM** as compared to PNIPAM only, with a molecular weight difference of 1313 Da, which is exactly the mass of BTA-mN_3_ (Figure S4). The number-averaged molar mass (*M*_n_) of **BTA-PNIPAM** and degree of polymerization (*DP*_n_) were calculated, which was ∼4200 Da and ∼42, respectively (Figures S1 and S3).

### Supramolecular Homoassembly of **BTA-PNIPAM** in the Dilute State

The solution and temperature-responsive behaviors of **BTA-PNIPAM** in Milli-Q (MQ) water were investigated in a dilute state using a combination of UV–vis spectroscopy, light scattering, and cryogenic transmission electron microscopy (cryo-TEM) (Figure 1B−1D). The cloud point temperature (*T*_cp_) of **BTA-PNIPAM** in MQ water (500 μM) was first determined by studying the UV–vis transmittance at 600 nm (Figure S5). A drop in transmittance to 50% of the initial value was denoted as the *T*_cp_. The *T*_cp_ of **BTA-PNIPAM** was 24 °C (Figure S5), which is 8 °C lower than that of free PNIPAM (32 °C). The BTA moiety, which comprises hydrogen bond donors and acceptors, is expected to interact with the PNIPAM segment. It is known that *T*_cp_ of PNIPAM is strongly associated with the structural changes in the proximal water molecules present near the PNIPAM chain.^60^ The inclusion of the BTA unit disrupts the interactions between PNIPAM and water, which promotes a faster dehydration of PNIPAM chains at lower temperatures. Temperature endurance cycles were measured to show recovery of **BTA-PNIPAM** after variations of temperature around *T*_cp_. Transmittance at 600 nm of the **BTA-PNIPAM** in MQ water (500 μM) was consistent, when the temperature fluctuated from 10 to 30 °C for 5 cycles (Figure S6). Since the onset temperature of the transmittance decrease was around 18 °C, all the following measurements were performed at 10 °C to ensure a hydrated state. The UV–vis spectrum of **BTA-PNIPAM** showed only the PNIPAM peaks at 208 and 310 nm, without the characteristic peak of the BTA fibrous structure at 227 nm,^31^ suggesting the absence of supramolecular polymerization (Figure 1B). SLS experiments (Figure 1C) revealed a slightly angle-dependent Rayleigh ratio (*R*) at low angles, similar to the PNIPAM precursor, which was a random coil at 10 °C.^35^ Moreover, the Rayleigh ratio was much smaller as compared to **BTA-EG**_**4**_, forming micrometer-long fibers itself (Figure S7),^27,29^ suggesting the formation of small aggregates for **BTA-PNIPAM**. DLS experiments with CONTIN fitting corroborated the presence of small nanostructures with a hydrodynamic diameter (*D*_h_) of ∼12 nm (Figure S8), while cryo-TEM confirmed the presence of spherical micellar structures with a diameter of ∼5–8 nm (Figure 1D). Altogether, the characterizations elucidated that **BTA-PNIPAM** cannot undergo supramolecular polymerization in the hydrated state, probably due to a too high hydrophilic/hydrophobic ratio or large shielding effect by the random coil PNIPAM chains, making it sterically difficult for the BTAs to self-assemble through intermolecular BTA-BTA interactions.

### Supramolecular Coassembly of BTA-PNIPAM in the Dilute State

**Figure 2:**
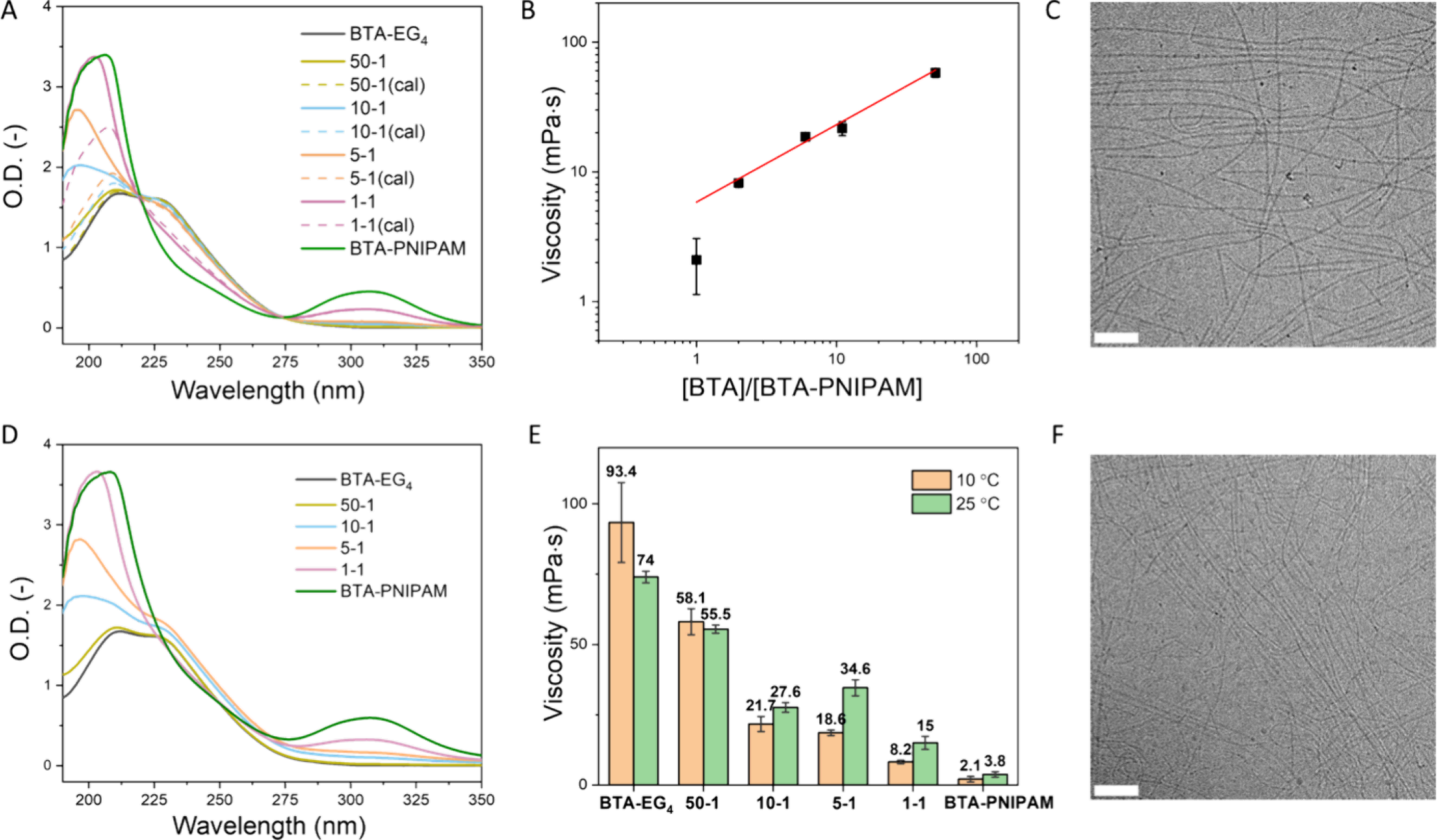
Characterization in the dilute state of coassemblies. UV–vis spectra of **BTA-EG**_**4**_ (50 μM), and mixtures of **BTA-EG**_**4**_ and **BTA-PNIPAM** ([BTA] = 50 μM) with different molar ratios in MQ-H_2_O with a cuvette path length of 1 cm at (A) 10 °C and (D) 25 °C. (B) Viscosity of **BTA-EG**_**4**_ and **BTA-PNIPAM** mixtures with a fixed concentration of 500 μM in MQ-H_2_O at 10 °C as a function of the reciprocal **BTA-PNIPAM** fraction (shear rate = 1 s^–1^). Cryo-TEM images of a mixture of **BTA-EG**_**4**_ (500 μM) and **BTA-PNIPAM** (100 μM) at (C) 10 °C and (F) 25 °C. Scale bars = 100 nm. (E) Viscosity ([BTA] = 500 μM) of **BTA-EG**_**4**_, **BTA-PNIPAM**, and their mixtures with different ratios in MQ-H_2_O at 10 and 25 °C (shear rate = 1 s^–1^). Data are represented as mean with SEM.

**BTA-PNIPAM** was subsequently introduced into **BTA-EG**_**4**_ solution at various molar ratios to investigate their interactions below and above its *T*_cp_, respectively. UV–vis spectra were recorded with a fixed BTA concentration of 50 μM and a series of molar ratios of **BTA-PNIPAM** and **BTA-EG**_**4**_ at 10 °C (Figure 2A). The signature peak around 227 nm decreased as the ratio of **BTA-PNIPAM** increased. Additionally, the measured spectra did not overlap with the calculated, self-sorted spectra (dotted calculated line), suggesting an interaction between **BTA-EG**_**4**_ and **BTA-PNIPAM**.^61^ On the contrary, if the measured coassembled absorbance spectrum would exactly overlap with the averaged sum of the individual homoassemblies (dotted calculated line), this would hint at a self-sorted state system. The *T*_cp_ of the **BTA-EG**_**4**_ and **BTA-PNIPAM** mixtures of equal total molar concentration (500 μM) indicated a less steep phase transition with a higher *T*_cp_ upon the increase of the **BTA-EG**_**4**_ ratio (Figure S9), also indicating the coassembled structures. Viscosities were subsequently measured to explore the length of the formed supramolecular polymers. Considering the possibility of shear-thinning and the surface tension effects on low-torque measurements,^62^ a shear rate of 1 s^–1^ was applied. Yet, the **BTA-EG**_**4**_ solution still showed rather big deviation, probably owing to the similar order of magnitude of the **BTA-EG**_**4**_ fiber length (micrometers long) and rheology gap height (56 μm) (Figure 2E). **BTA-PNIPAM** was effective to reduce the **BTA-EG**_**4**_ solution viscosity from 90 to 2 mPas at 10 °C. The viscosity displays a power law relationship with a power law exponent of ∼0.6 as a function of the reciprocal **BTA-PNIPAM** fraction (Figure 2B). The Cates model (also confirmed experimentally) predicts that this scaling exponent corresponds to a fast-breaking mechanism of the supramolecular polymer chains. Thus, **BTA-PNIPAM** was most likely acting as a chain capper or sequestrator: **BTA-PNIPAM** destabilized and disrupted the **BTA-EG**_**4**_ fibers—shortening the fibers.^63,64^ Cryo-TEM was in line with the UV and viscosity results, showing short fibers at a ratio of 5/1 for **BTA-EG**_**4**_**/BTA-PNIPAM** (Figure 2C). Once the temperature was increased above the *T*_cp_ to 25 °C, the PNIPAM chains were gradually dehydrated and thereby served as a cross-linker to enable the elongation and conjunction of short fibers. Thus, the fibrous signature peak at 227 nm increased along with slightly increased scattering in the UV–vis spectra (Figure 2D and Figure S10). The viscosity of the mixtures also increased at higher **BTA-PNIPAM** fractions (Figure 2E), while cryo-TEM showed fibers that were much longer in comparison with the hydrated state (Figure 2F).

### Hybrid, Supramolecular Hydrogel: Mechanical and Dynamic Characterization

The thermoresponsive mechanical properties of the hybrid, supramolecular hydrogels consisting of mixtures of **BTA-EG**_**4**_ and **BTA-PNIPAM** at mM concentrations were investigated using rheology. Briefly, samples were prepared with a fixed **BTA-EG**_**4**_ concentration (7.8 mM, 1 wt %), at which **BTA-EG**_**4**_ fibers were entangled into a 3D network to form a weak hydrogel (**G-0** hydrogel). A series of mixtures were generated in the presence of 1 wt % **BTA-EG**_**4**_ with additionally added: 0.1, 0.5, or 1 wt % of **BTA-PNIPAM** with corresponding molar ratios between **BTA-EG**_**4**_/**BTA-PNIPAM** of ∼50/1, 10/1, and 5/1, to afford hydrogels **G-0.1**, **G-0.5**, and **G-1**, respectively. Importantly, these molar ratios between **BTA-EG**_**4**_ and **BTA-PNIPAM** match with the measured coassembled solutions in the dilute state above.

**Figure 3:**
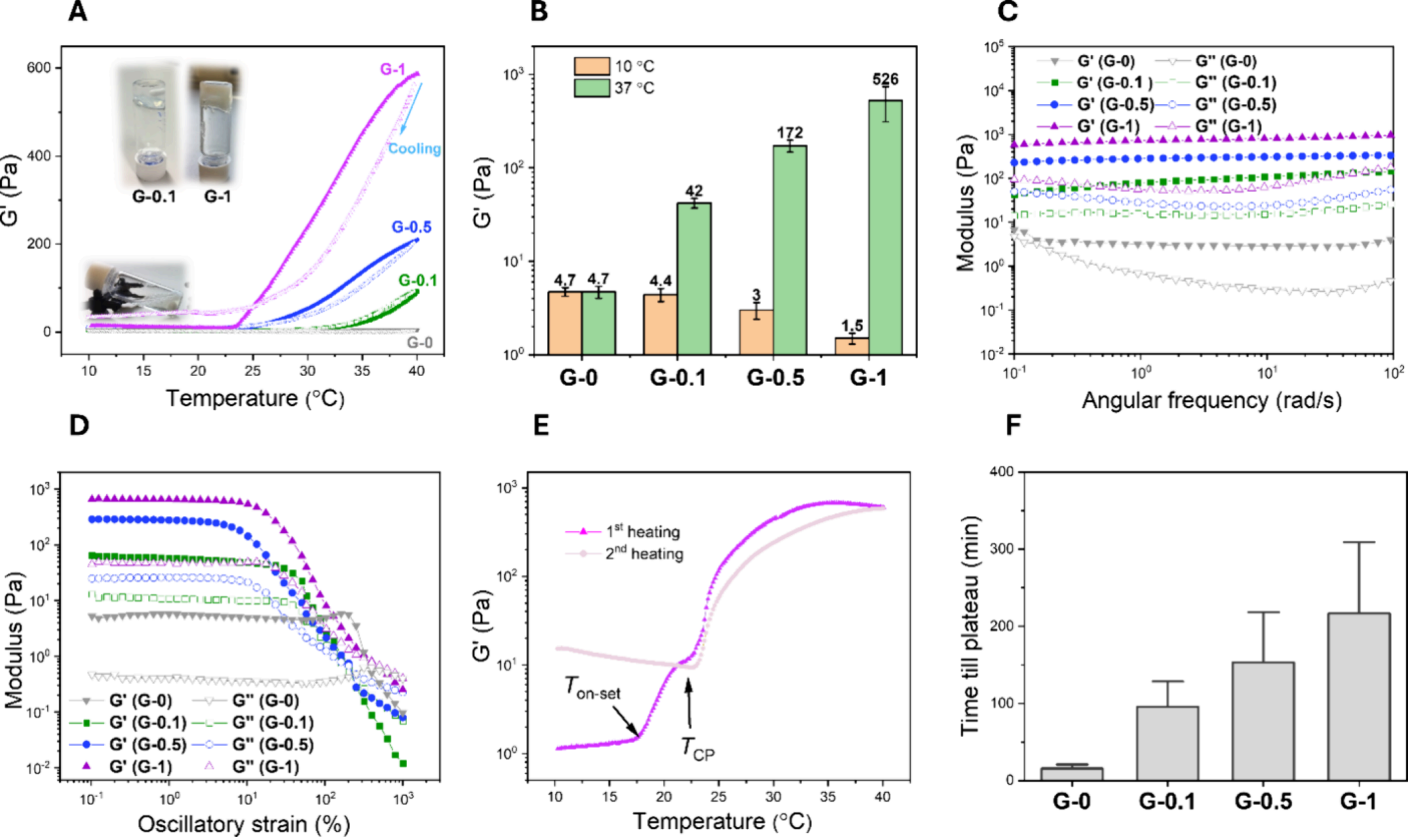
Mechanical and dynamic characterization of thermoresponsive, hybrid supramolecular hydrogels. (A) Storage (*G*′) and loss (*G*′′) moduli vs temperature at a strain of 1%, an angular frequency of 1 rad/s, and a heating/cooling rate of 1 °C/min for the mixtures of **BTA-EG**_**4**_ and **BTA-PNIPAM**. The concentration of **BTA-EG**_**4**_ was fixed as 1 wt %, and **BTA-PNIPAM** was varied from 0, to 0.1, to 0.5, and to 1 wt %, to afford **G-0**, **G-0.1**, **G-0.5**, and **G-1**, respectively. Insets: photographs of (below) a vial containing **G-1** (solution) at 10 °C, and (above) inverted vials containing **G-0.1** (transparent hydrogel) and **G-1** (opaque hydrogel) at 37 °C. (B) *G*′ of **G-0**, **G-0.1**, **G-0.5**, and **G-1** at 10 and 37 °C, respectively. (C) Angular frequency (applied strain = 1%) and (D) oscillatory strain (angular frequency = 1 rad/s) sweeps of **G-0, G-0.1**, **G-0.5**, and **G-1** at 37 °C. (E) Thermoresponsive mechanical properties of **G-1** in the first and second heating scans. Arrows indicate the onset dehydration temperature and *T*_cp_ of **BTA-PNIPAM**. (F) Quantified FRAP of **G-0**, **G-0.1**, **G-0.5**, and **G-1**, showing the time when fluorescence intensity reaches plateau (*n* = 3). All samples contain 20 μM of **BTA-Cy5**. Quantified data are represented as mean with SEM.

Rheology at 10 °C revealed that, although the storage modulus *G*′ remained larger than the loss modulus *G*″ throughout all four samples, the mixtures existed as flowing viscous liquids with quite low *G*′ of 1–5 Pa (Figure 3A,B). Temperature sweeps were performed with a heating rate of 1 °C/min, which showed increasing stiffness for all three hybrid hydrogels above the *T*_cp_ of **BTA-PNIPAM** (Figure 3A,B). A constant modulus of the control **G-0** hydrogel without **BTA-PNIPAM** was observed upon increasing the temperature (Figure 3A,B). The transitions from viscous liquids to a transparent weak hydrogel (*G*′ ∼42 Pa for **G-0.1**) or opaque stiff hydrogels (*G*′ ∼172 Pa for **G-0.5** and *G*′ ∼526 Pa for **G-1**) were observed as temperature increased (Figure 3B,C). Among these hybrid hydrogels, **G-1** possessed the highest *G*′ of ∼526 Pa at 37 °C, with an increase in stiffness, which was more than 2 orders of magnitude as compared to the **G-0** hydrogel with *G*′ ∼5 Pa (Figure 3B–D). This suggests that the higher concentration of **BTA-PNIPAM** could ensure a higher degree of cross-linking within the hydrogel—a behavior that was also observed in the dilute state with viscosity and cryo-TEM experiments (Figure 2E,F). Moreover, oscillation strain (Figure 3C) and angular frequency (Figure 3D) sweeps were performed on the three hybrid hydrogels at 37 °C. The moduli of the hydrogels were all dominated by the elastic contributions at the applied frequencies. However, the two samples with the highest **BTA-PNIPAM** concentrations (**G-0.5** and **G-1**) contained a higher elastic modulus, which was frequency-independent, whereas the hydrogel with the lowest **BTA-PNIPAM** content (**G-0.1**) showed clear relaxation in the low frequency range (Figure 3C). The **BTA-PNIPAM** thus clearly altered the stiffness and dynamics of the hybrid networks. Noteworthy, the increased stiffness of the hybrid hydrogel does not cause a significant reduction of the linear viscoelastic region, as indicated by the strain sweep (Figure 3D). While the strain at which the *G*′ – *G*′′ crossover occurred, varied based on the **BTA-PNIPAM** content, both **G-0.1** and **G-0.5** displayed a *G*′ – *G*′′ crossover around ∼250% strain (Figure 3D). **G-1** displayed a remarkable breakdown strain appearing at 500% (Figure 3D), probably owing to a high cross-linking content.

Zooming in on the temperature sensitivity of the different hydrogels, the rigidification of hydrogel **G-1** took place at the onset dehydration temperature of **BTA-PNIPAM** and underwent another steeper increase of *G*′ at *T*_cp_ (Figure 3E). However, the second temperature sweep did not overlap with the first one, only showing the stiffening onset at *T*_cp_ with additionally a higher initial *G*′. Moreover, the apparent *G*′ from the first sweep appeared to decrease after it reached a maximum at ∼35 °C. Additionally, the cooling curves were not fully reversible (Figure 3A). **G-0.1** displayed a slight hysteresis, while **G-0.5** and **G-1** showed an immediate and sharp decrease of *G*′ when the sample was cooled down. Additionally, it was noticed that all of the hybrid hydrogels showed syneresis (i.e., water being expelled from the gel) when incubated at 37 °C overnight, with visible macroscopic shrinkage as well as water being expelled from the hydrogels. While at room temperature (20 °C) over several weeks, no macroscopic shrinkage was observed, nor water expulsion from the hydrogels was observed. The sharp drop during the cooling process of **G-0.5** and **G-1** is hypothesized to occur due to the syneresis associated with these hydrogels, which introduced phase separation and altered hydrogel inner structures upon heating (Figure S11). The opaque color further suggests the occurrence of phase separation and possibly irreversible structural changes in the gel upon heating–cooling.

Additionally, controls containing physical mixtures of PNIPAM and **BTA-EG**_**4**_ were investigated on their thermoresponsive mechanical properties (Figure S12). The physical mixtures contain identical ratios between PNIPAM and **BTA-EG**_**4**_ as well as similar concentrations (wt %) to networks that contain the covalently attached **BTA-PNIPAM**. Only the mixture with the highest amount of PNIPAM (1 wt %) showed a slight sol–gel transition upon heating. However, this physical mixture was still ∼15 times softer (*G*′ ∼34 Pa) as compared to the network that contained the covalently attached **BTA-PNIPAM** (*G*′ ∼526 Pa), along with a profound syneresis effect (Figure 3B and Figure S11). This shows that the networks containing covalently attached **BTA-PNIPAM** clearly outperform the physically mixed PNIPAM and **BTA-EG**_**4**_ networks and highlight the need for the covalent attachment of PNIPAM to BTA.

Next, the dynamics of the hybrid hydrogels at 37 °C were investigated using FRAP. **BTA-Cy5** (20 μM) was included in the hydrogels for visualization (Figure 1A). A laser at 633 nm at the maximum laser power was used to bleach the fluorophores in the region of interest (ROI, <10% of total image area). Diffusion of both the monomers within the supramolecular fibers and the fibers within the hydrogel^37^ will in time result in replacement of the bleached fluorophores in the ROI by nonbleached fluorophores, which were initially located outside the ROI.

A homogeneous, red-colored image was only observed for **BTA-EG**_**4**_ (Figure S11A), while an increase in domain formation was revealed for higher **BTA-PNIPAM** contents in the hydrogels (Figure S11B–D). We hypothesize that higher **BTA-PNIPAM** contents in the hydrogels result in a higher tendency of the hydrophobic domains to collapse and will thus lead to phase separation and a more heterogeneous structure. Connecting and comparing these FRAP results, which show the microphase separation to the UV–vis measurements, which suggested coassembly, rather than self-sorting, it should be realized that there is a big difference in concentration (gel state ∼mM concentration versus dilute state ∼μM concentration, respectively) and analyzed sample size of the measurement (∼μm scale versus cm scale, respectively).

Subsequently, the recovery of the fluorescence signal was then tracked for up to a maximum of 6 h (Figure 3F). The **G-0** showed quick recovery with a half-life (τ_1/2_) during which the fluorescence intensity recovered to half its original value of ∼7 min, contained a diffusion constant (*D*) of ∼1.5 μm^2^/s, and reached plateau after 15 min, with an immobile fraction of ∼0.5 (Figure 3F, Figures S13A and S14). Full recovery of the fluorescence signal could not be reached within the measured time frame of 6 h. It should be realized that the sample contains entangled, μm-long fibers,^31^ of which the length is smaller than the total bleached area (50 μm), such that the fibers might not fully diffuse out of the bleached area during the time frame of the experiment. The samples containing 0.1, 0.5, and 1 wt % **BTA-PNIPAM** all showed slower recovery compared to **BTA-EG**_**4**_ (Figure S13B–D), with a τ_1/2_ of ∼40, 51, and 37 min, contained a *D* of ∼0.3, 0.5, and 0.4 μm^2^/s (Figure S14) and reached plateau after 100, 153, and 216 min of measuring (Figure 3F), respectively. Noteworthy, τ_1/2_ was slower (∼51 min) for **G-0.5** than for **G-1** (∼37 min), which might result from the heterogeneity/phase separation of the samples. Moreover, the total concentration of BTA is not equal in these samples (1.5 wt % for **G-0.5** versus 2 wt % in **G-1**, while maintaining the **BTA-EG**_**4**_ identical), which might influence the dynamic behavior of the hydrogels.

Together, these data reveal a clear trend; at 37 °C, the diffusion is hindered more upon increasing the content of **BTA-PNIPAM** in the supramolecular hydrogels, indicating slower dynamics. We hypothesize that this is caused by the tendency of PNIPAM to collapse its hydrophobic domain, leading to more domain formation in which both the **BTA-Cy5** monomers inside the fibers as well as the **BTA-Cy5** containing fibers within the whole gel could diffuse less freely.

### Cell Encapsulation inside Hybrid Supramolecular Hydrogel through Sol–Gel Transition

**Figure 4:**
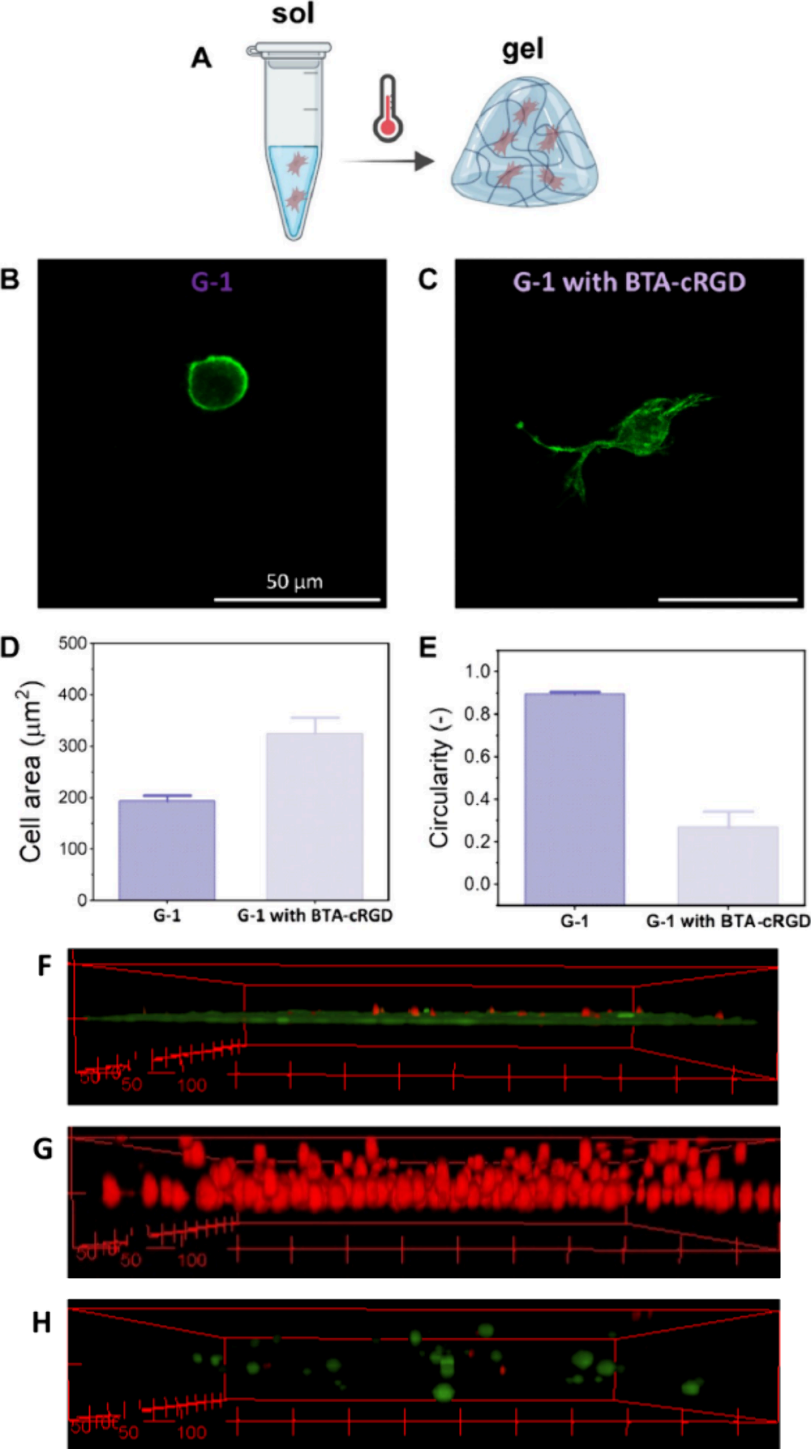
Facile cell encapsulation in 3D inside hybrid supramolecular hydrogels through temperature-responsive sol–gel transition (A). (B, C) Cell culture of human normal dermal fibroblasts (hNDF) inside supramolecular hydrogels containing 1 wt % **BTA-EG_4_** and 1 wt % **BTA-PNIPAM** (G-1), with (**G-1** with **BTA-cRGD**) and without (**G-1**) the cell binding supramolecular monomers **BTA-cRGD**. [**BTA-cRGD**] = 1 mM. Scale bar = 50 μm. One day culture, green = F-actin. (D, E) Quantification of cell area and circularity of cell cultured inside the thermoresponsive supramolecular hydrogels. *n* = 10 individual cells per condition. Data are represented as mean with SEM (F–H) 3D-live/dead staining images (green = live, red = dead) of a positive control without hydrogel (F), a negative control with 70% ethanol (G), and cells encapsulated in hydrogel **G-1** with **BTA-cRGD** (H) after 24 h. Images are constructed of 21 images every 5 μm along the *z*-axis. Scale bar = 50 μm/tick.

The temperature responsiveness of the hybrid supramolecular hydrogels enables facile cell encapsulation at low temperatures in solution and the formation of a gel upon increasing the temperature. Fibroblast cells were encapsulated inside the solution at low temperatures as single cells, and upon increasing the temperature to 37 °C, the formation of a gel with cells encapsulated inside was achieved (Figure 4A). Cell viability was determined using an MTT assay (Figure S16). None of the hydrogels exhibited toxic effects to the cells in the measured 24 h time frame, as cell viability was greater than 85% for all conditions. Cell morphology was then analyzed with and without 1 mM of the integrin-binding moiety **BTA-cRGD**(37) (Figure S17) present inside the hydrogels (**G-1 with BTA-cRGD**), or not (**G-1**). For these experiments, the total concentration of **BTA-EG**_**4**_ was kept constant, meaning that the included amount of **BTA-cRGD** was extracted from the total 1 wt % **BTA-EG**_**4**_, leading to slightly less [**BTA-EG**_**4**_] in the gels containing **BTA-cRGD** as compared to the gels lacking **BTA-cRGD** (see Sample Preparation). Cell spreading was observed inside the hydrogels containing **BTA-cRGD** and not when **BTA-cRGD** was absent (Figure 4B,C and Figure S18). This was also reflected in the lower circularity and higher cellular spreading area for the cells inside the **BTA-cRGD** containing hydrogels (**G-1 with BTA-cRGD**) as compared to those inside the bare hydrogels (**G-1 without BTA-cRGD**, Figure 4D,E). This shows that (1) 3D cell encapsulation is possible and (2) that the cell adhesion motif **BTA-cRGD** is required to achieve cell spreading. However, when comparing the cell spreading area using similar cell type (fibroblasts) inside this supramolecular hydrogel (3D culture) to that on other BTA-based supramolecular hydrogels (2D culture), cell spreading area is still marginal (here, 300 μm^2^ versus up to 500 μm^2^ for the 2D culture).^37^ Possible explanations for these differences include the difference in culture dimensionality, with cells encapsulated in 3D possibly experiencing hindrance from the small pore sizes and/or mechanical constriction inside the network. Also, a profound syneresis effect was observed for these hybrid hydrogels when incubated at 37 °C for longer periods (i.e., overnight), possibly resulting in local stiff and dense areas. This might affect the cell spreading area. Furthermore, live/dead staining was executed on cells encapsulated in the hydrogel **G-1** with **BTA-cRGD** for 24 h (Figure 4F–H and Figure S19). These results are consistent with the cell viability study and show a majority of living cells. Also, all cells are well separated from each other and homogeneously distributed compared to the positive control, resulting in only cell–matrix interactions.

## Conclusions

In this investigation, we expanded the responsiveness of the BTA-based supramolecular system by introducing temperature sensitivity, which provides opportunities as synthetic ECM with quick and facile performance. The temperature-responsive **BTA-PNIPAM** serves as a chain capper, shortening the fibers, below *T*_cp_ and as a cross-linker above *T*_cp_. This allows for tunability and control over the mechanical and dynamic properties as a function of the temperature using a modular strategy. In the gel state, both transparent weak hydrogels with high dynamic and opaque stiff hydrogels with low dynamics were obtained. Facile 3D cell encapsulation was realized, and integrin-binding motifs were required to achieve cell spreading. A profound syneresis effect was, however, observed for these hydrogels when incubated at 37 °C for prolonged periods, resulting in locally dense and stiff areas, which might hamper cell spreading. More studies are ongoing to better reveal the phase separation effect toward cell behavior. Moreover, the syneresis effect, capable of maintaining hydrogel shape, might benefit the shrinking-orientated 3D-printed tissue engineering.^65−67^

## Supporting Information Available

Synthetic routes for compounds **PNIPAM** and **BTA-PNIPAM**, including characterization with ^1^H NMR, FT-IR and MALDI-TOF MS spectra, SEC chromatogram, Cryo-TEM, transmittance and dynamic light scattering measurements, MTT assay, rheology measurement, additional cell images, and further dynamic characterization using FRAP; chemical structure of **BTA-cRGD**(37) (PDF)
