# Supplementary material for: Molecularly Engineered Supramolecular Thermoresponsive Hydrogels with Tunable Mechanical and Dynamic Properties
Source: Biomacromolecules. 2024 Jul 26;25(8):4686–96. doi: 10.1021/acs.biomac.3c01357 (PMC11323010; doi:10.1021/acs.biomac.3c01357)
Supplement: Supplementary file 1 — bm3c01357_si_001.pdf [file bm3c01357_si_001.pdf]

Supplementary information

Molecularly Engineered Supramolecular Thermoresponsive Hydrogel with Tunable Mechanical and Dynamic Properties

*Laura Rijns[1], Heleen Duijs[2], René P. M. Lafleur[3], Ruth Cardinaels[4,5], Anja R. A. Palmans[3], Patricia Y. W. Dankers[1] and Lu Su[2]**

[1]Department of Biomedical Engineering, Institute for Complex Molecular Systems (ICMS), Eindhoven University of Technology, 5600 MB Eindhoven, The Netherlands.

[2]Leiden Academic Centre for Drug Research (LACDR), Leiden University, Wassenaarseweg 76, 2333 AL Leiden, The Netherlands.

[3]Laboratory of Macromolecular and Organic Chemistry, Institute for Complex Molecular Systems (ICMS), Department of Chemical Engineering and Chemistry, Eindhoven University of Technology, 5600 MB Eindhoven, The Netherlands.

[4]Processing and Performance of Materials, Institute for Complex Molecular Systems (ICMS), Department of Mechanical Engineering, Eindhoven University of Technology, 5600 MB Eindhoven, The Netherlands.

[5]Soft Matter, Rheology and Technology, Department of Chemical Engineering, KU Leuven, 3001 Leuven, Belgium.

Table of contents

Synthesis: Scheme S1

Supporting Figures: Figure S1-S19

References

Synthesis

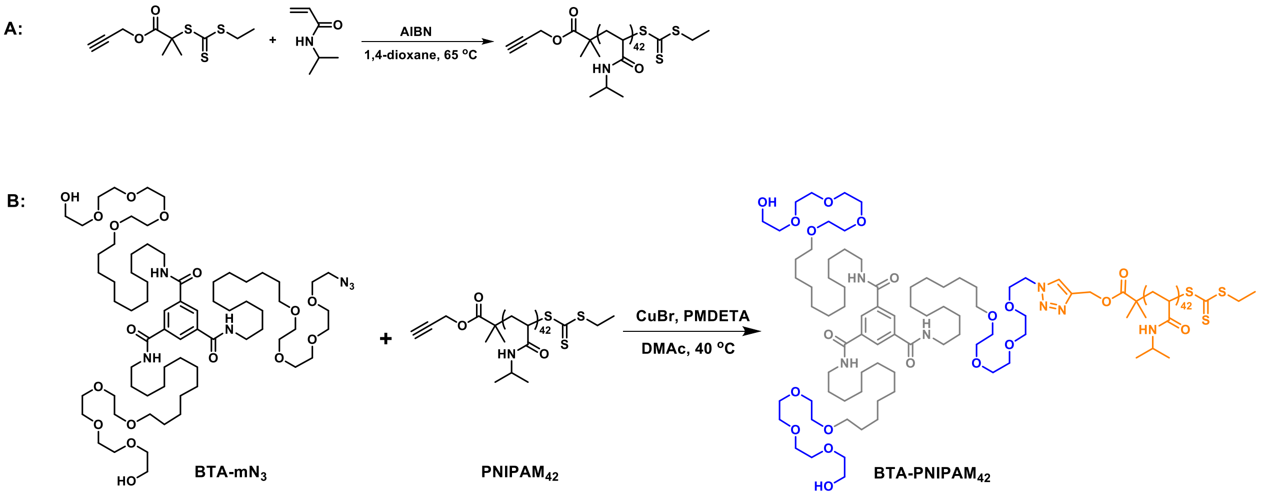

Scheme S1: **Synthetic route towards BTA-PNIPAM**. (**A**) Chemical synthetic route to afford alkyne functionalized PNIPAM *via* RAFT polymerization. (**B**) Synthetic route to afford **BTA-PNIPAM** through an azide-alkyne Huisgen cycloaddition reaction between alkyne functionalized PNIPAM and mono-azide BTA precursor (**BTA-mN_3**) with overall yield of 60.4 %.

Supporting Figures

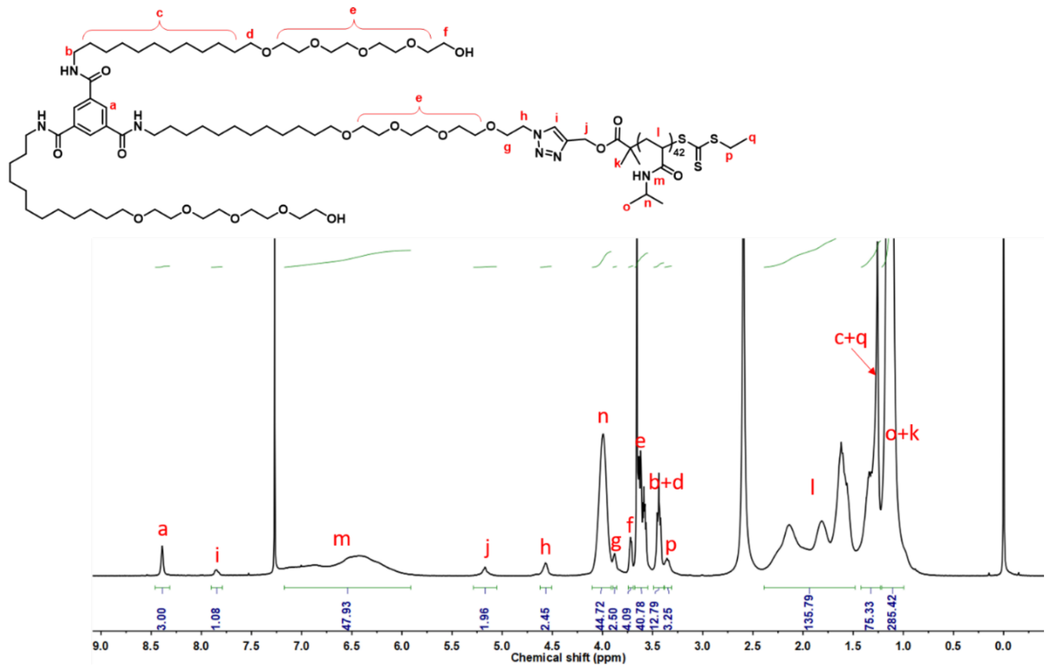

Figure S1: ^{1}H NMR of BTA-PNIPAM in $CDCl_3$ (400 MHz) with all peaks assigned.

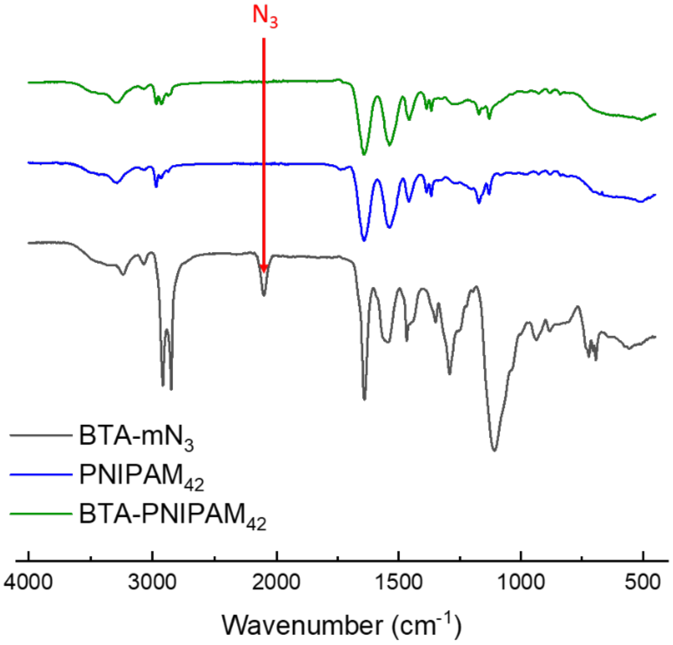

Figure S2: **FT-IR spectra of BTA-EG$_4$, PNIPAM and BTA-PNIPAM**, showing the disappearance of N_3 peak in 2110 in **BTA-PNIPAM**. The N_3 asymmetric stretch of the azide group is marked.

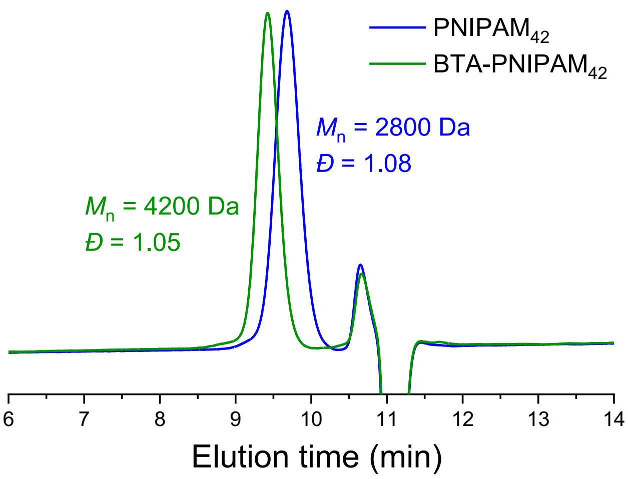

Figure S3: **SEC chromatograms (DMF as eluent, 1.0 mL/min) of PNIPAM and BTA-PNIPAM**.

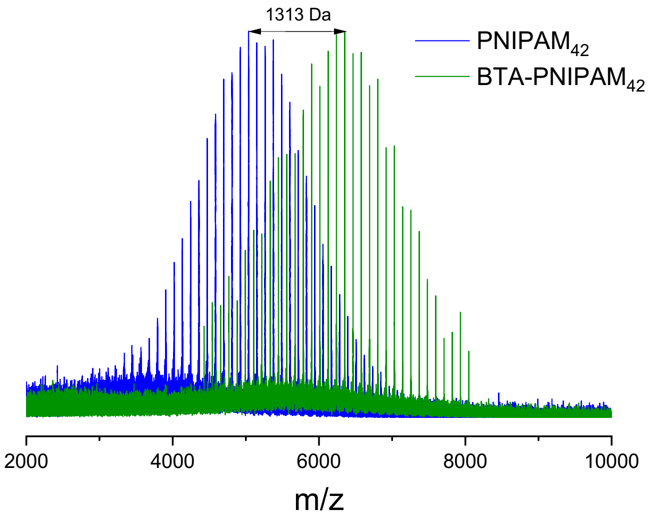

Figure S4: **MALDI-TOF MS spectra of PNIPAM and BTA-PNIPAM**, with the difference of the two population to be 1313, exactly the MW of BTA-mN_3.

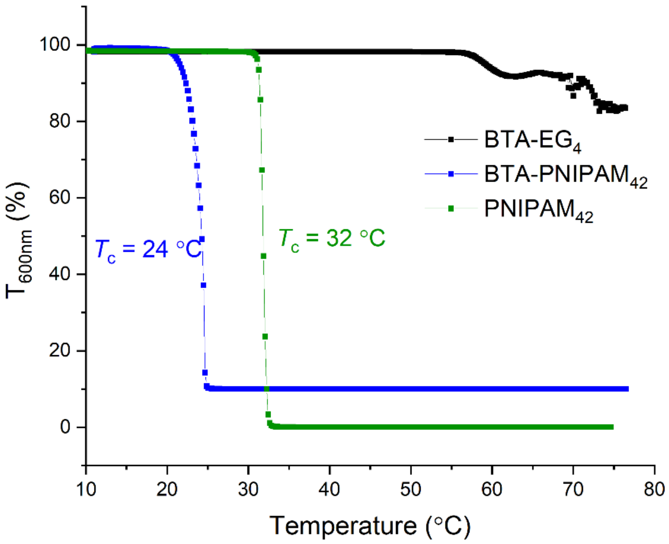

Figure S5: **Transmittance at 600 nm of BTA-EG$_4$ (500 µM), BTA-PNIPAM (500 µM), and PNIPAM (500 µM),** recorded at different temperatures. The cloud points of **BTA-EG$_4$**, **BTA-PNIPAM**, and PNIPAM are *ca*. 57 °C, 24 °C, and 32 °C respectively.

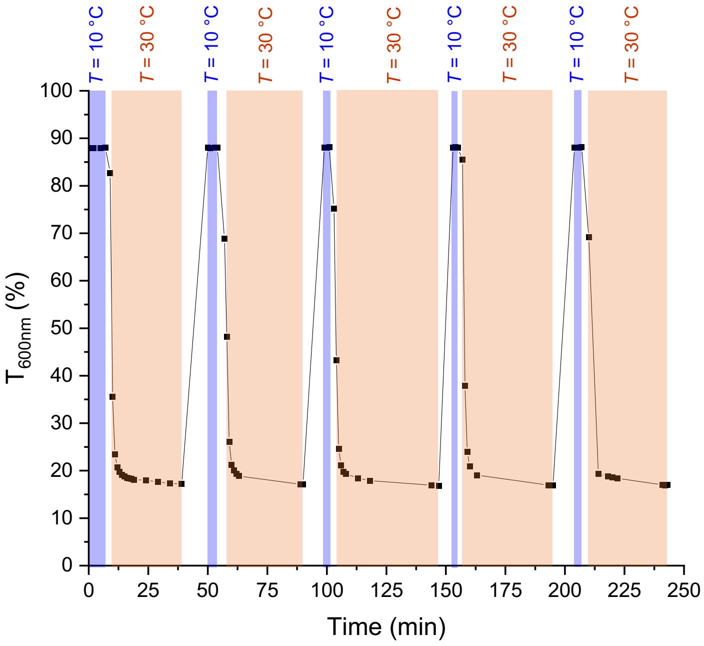

Figure S6: **Transmittance at 600 nm of BTA-PNIPAM (500 µM) at 10 °C and 30 °C for 5 cycles. BTA-PNIPAM** was heated to 30 °C after which the sample was allowed to equilibrate for 30 min. After cooling to 10 °C, the transmittance stabilized within 1 min to 88%. Furthermore, the cuvette was subjected to a stream of N_2 gas to prevent condensation on the glass.

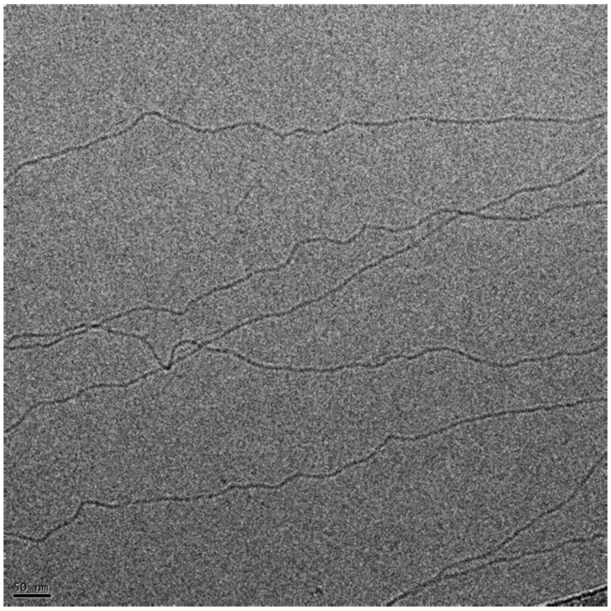

Figure S7: **CryoTEM image of BTA-EG$_4$** (500 µM) in MQ water at 22 °C, showing long fibers without ends. Scale bar = 50 nm. The black spherical particles are ice-crystals.

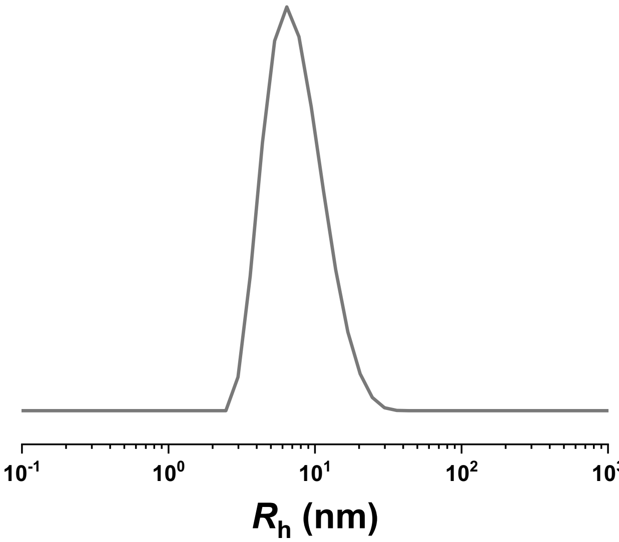

Figure S8: **Hydrodynamic radius (*R*$_h$) of ~ 6 nm for BTA-PNIPAM** (500 µM) in MQ water at 10 °C, as measured by dynamic light scattering.

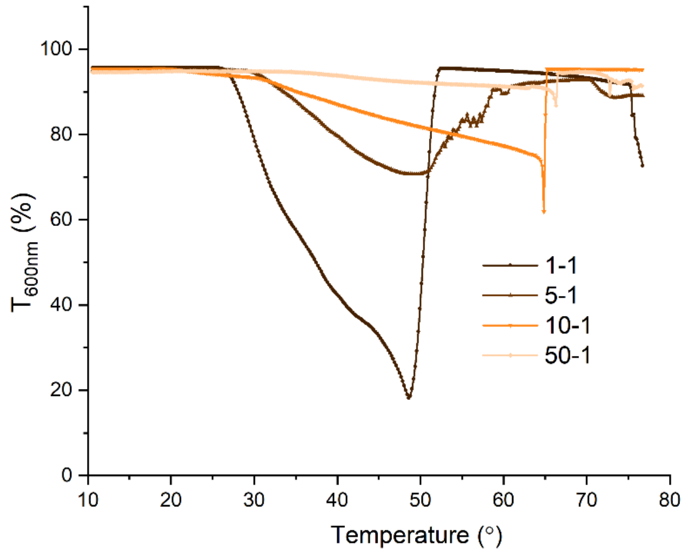

Figure S9: **Transmittance at 600 nm of the mixture of BTA-EG$_4$ and BTA-PNIPAM at varied molar ration ([BTA] = 500 µM),** recorded at different temperatures. The mixtures all showed a second sharp phase transition and the $T_{600\ nm}$ (%) recovered to be almost 100%, indicating a macroscopic aggregation.

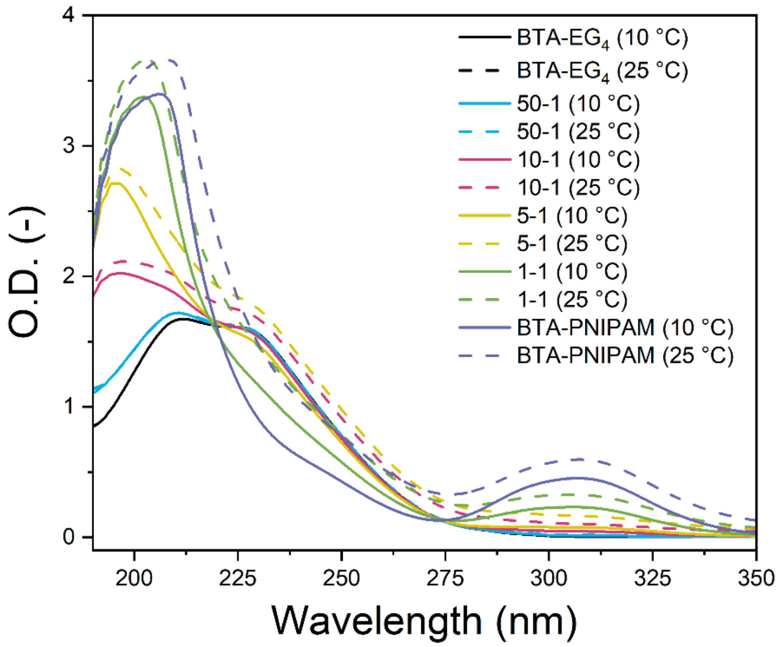

Figure S10. UV-vis spectra of BTA-EG$_4$ (50 µM), and mixtures of **BTA-EG$_4$** and **BTA-PNIPAM** ([BTA] = 50 µM) with different molar ratios in MQ-H_2O with a cuvette pathlength of 1 cm at both 10 °C and 25 °C.

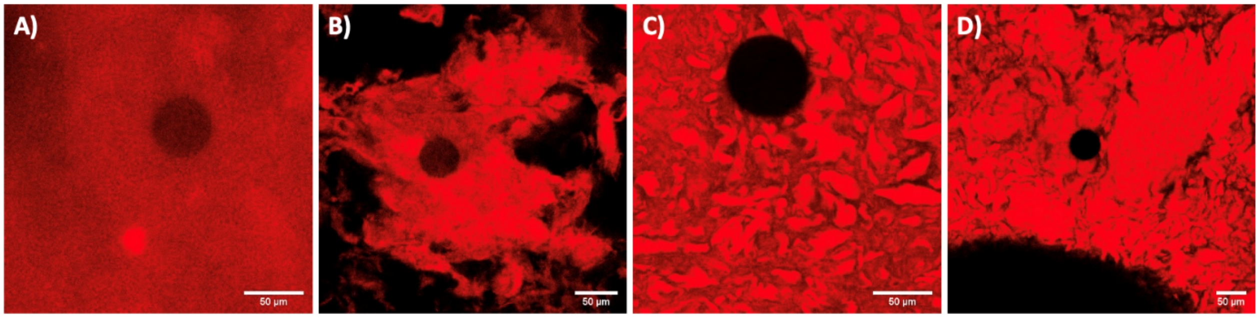

Figure S11: **Fluorescence confocal microscopy images directly after photobleaching** of (**A**) **BTA-EG$_4$**, (**B**) **BTA-EG$_4$** with 0.1 wt% **BTA-PNIPAM**, (**C**) **BTA-EG$_4$** with 0.5 wt% **BTA-PNIPAM** and (**D**) **BTA-EG$_4$** with 1 wt% **BTA-PNIPAM**. All samples contain 1 wt% **BTA-EG$_4$** and 20 µM **BTA-Cy5**. Scale bar = 50 µm.

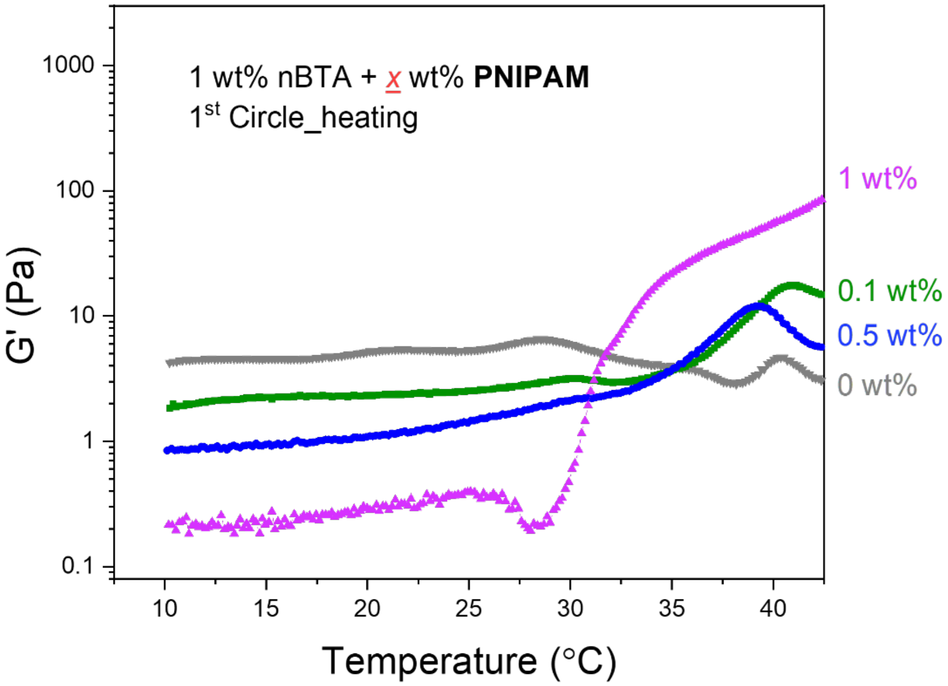

Figure S12: **Thermo-responsive mechanical characterization of mixtures of BTA-EG$_4$ and PNIPAM.** Storage (G') and loss (G") moduli *vs* temperature at a strain of 1%, an angular frequency of 1 rad/s, and a heating/cooling rate of 1 °C/min for the mixtures of **BTA-EG$_4$** and PNIPAM. The concentration of **BTA-EG$_4$** was fixed as 1 wt% and PNIPAM was varied from 0, to 0.1, to 0.5 to 1 wt%.

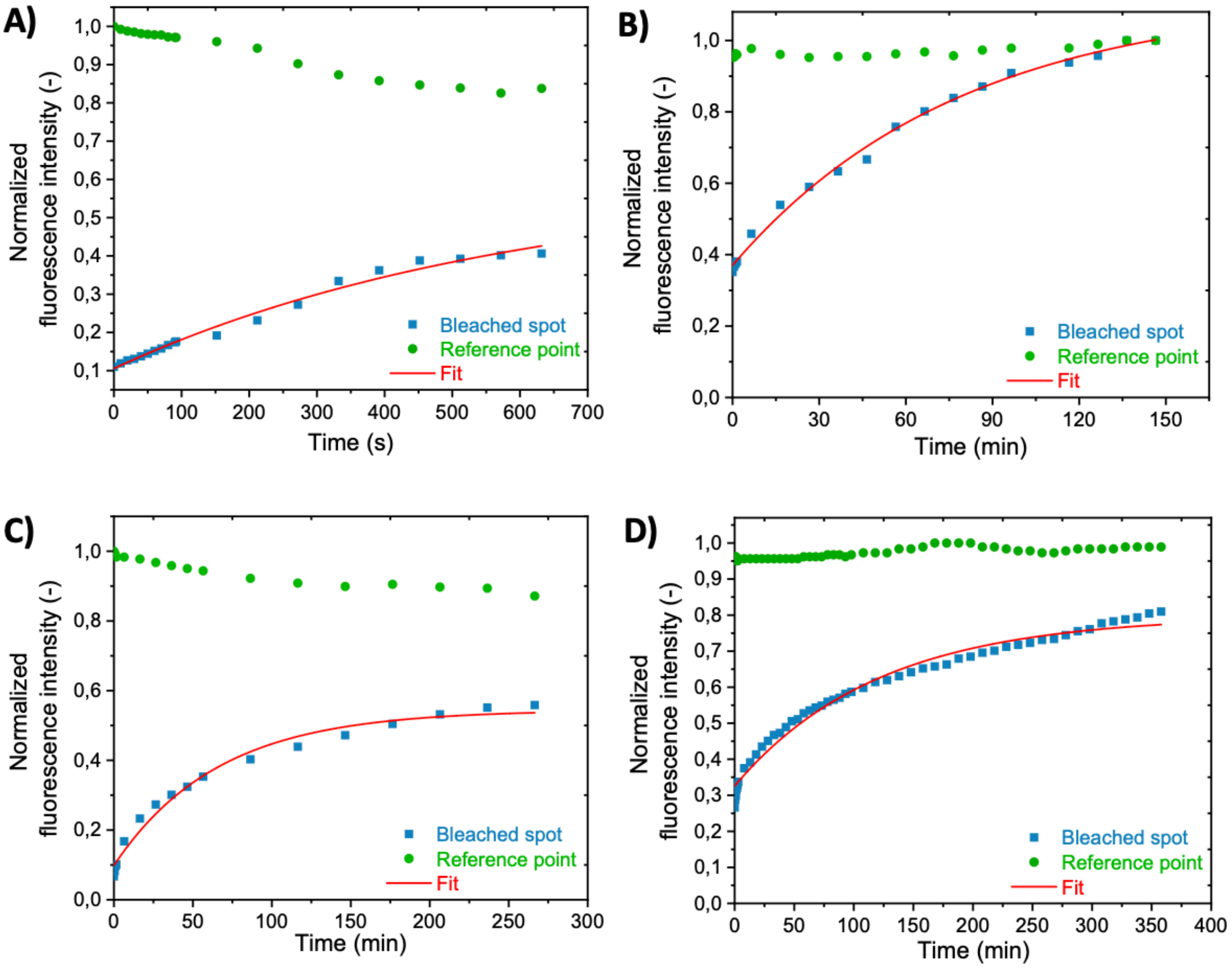

Figure S13: (**A-D**) **Fluorescence recovery after photo-bleaching (FRAP) experiments performed on hydrogels containing** (**A**) 1 wt% **BTA-EG$_4$** (**G-0**), (**B**) 1 wt% **BTA-EG$_4$** with 0.1 wt% **BTA-PNIPAM** (**G-0.1**), (**C**) 1 wt% **BTA-EG$_4$** with 0.5 wt% **BTA-PNIPAM** (**G-0.5**) and (**D**) 1 wt% **BTA-EG$_4$** with 1 wt% **BTA-PNIPAM** (**G-1**). All samples contain 20 µM of **BTA-Cy5**. Fluorescence recovery in the bleached spot is fitted by a single exponential growth model.

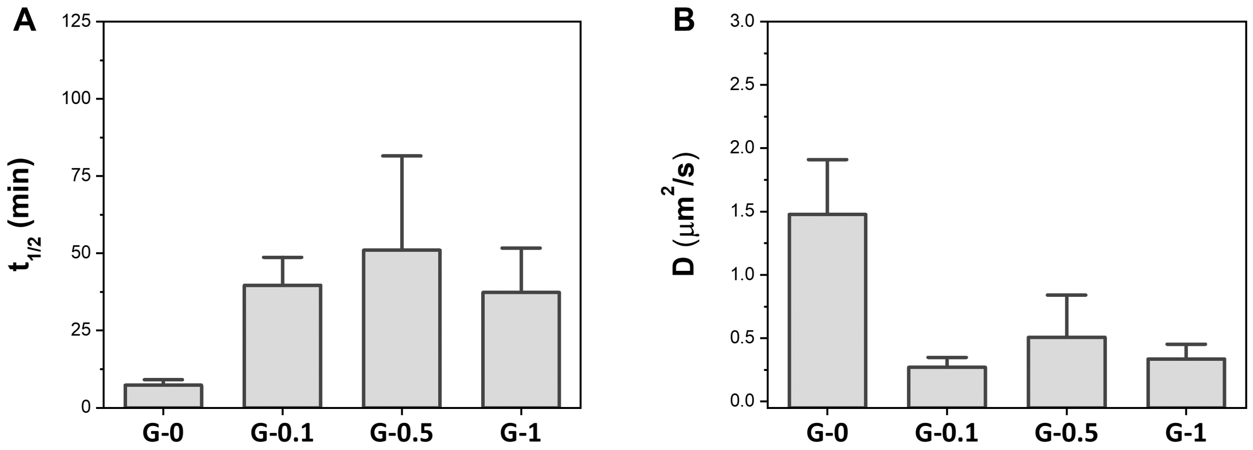

Figure S14: **Quantified FRAP results,** showing (**A**) the fluorescence half-life ($\tau_{1/2}$) during which the fluorescence intensity recovered to half its original value and (**B**) the extracted diffusion constant (D) in $\mu m^2/s$. For all quantified data holds n=3 and data are represented as mean with SEM.

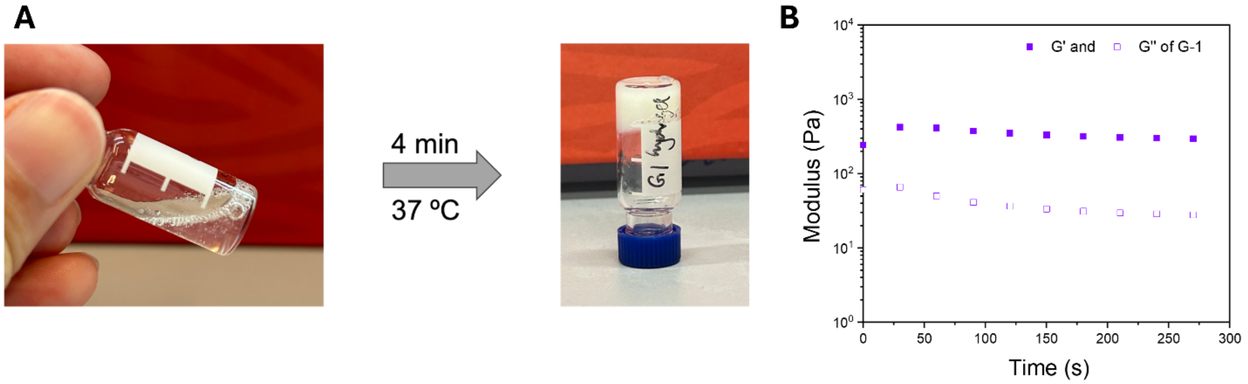

Figure S15: (**A**) The hydrogel **G-1** undergoes sol to gel transition at 37 °C within 4 min, as indicated by the vial inversion test. (**B**) Sample **G-1** was loaded in its liquid state onto the rheometer at 10 °C, after which the temperature was switched to 37 °C (which takes less than 1 min) to initiate gelation and a time sweep was measured at 1% strain and 1 rad/s. t_0 equals the moment that the rheometer reached 37 °C, and a gel was already formed, which remained stable.

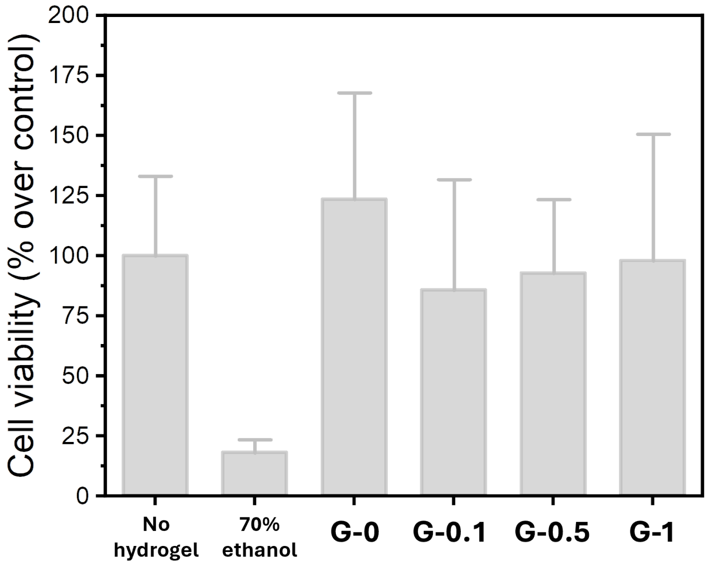

Figure S16: Cell viability study of G-0, G-0.1, G-0.5 and G-1 gels as determined by MTT assay. hNDF were cultured inside the gels for 24 h. Viability is normalized against cells cultured without hydrogels (*i.e.* no hydrogel) and cells treated with 70% ethanol were used as negative control. Data is plotted as mean with S.D., with n=6 per condition.

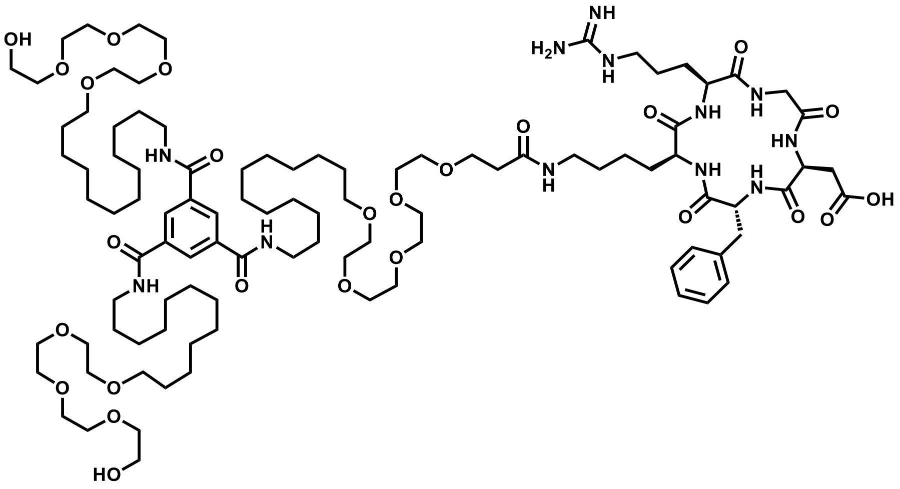

Figure S17: **Molecular structure of BTA-cRGD**[2].

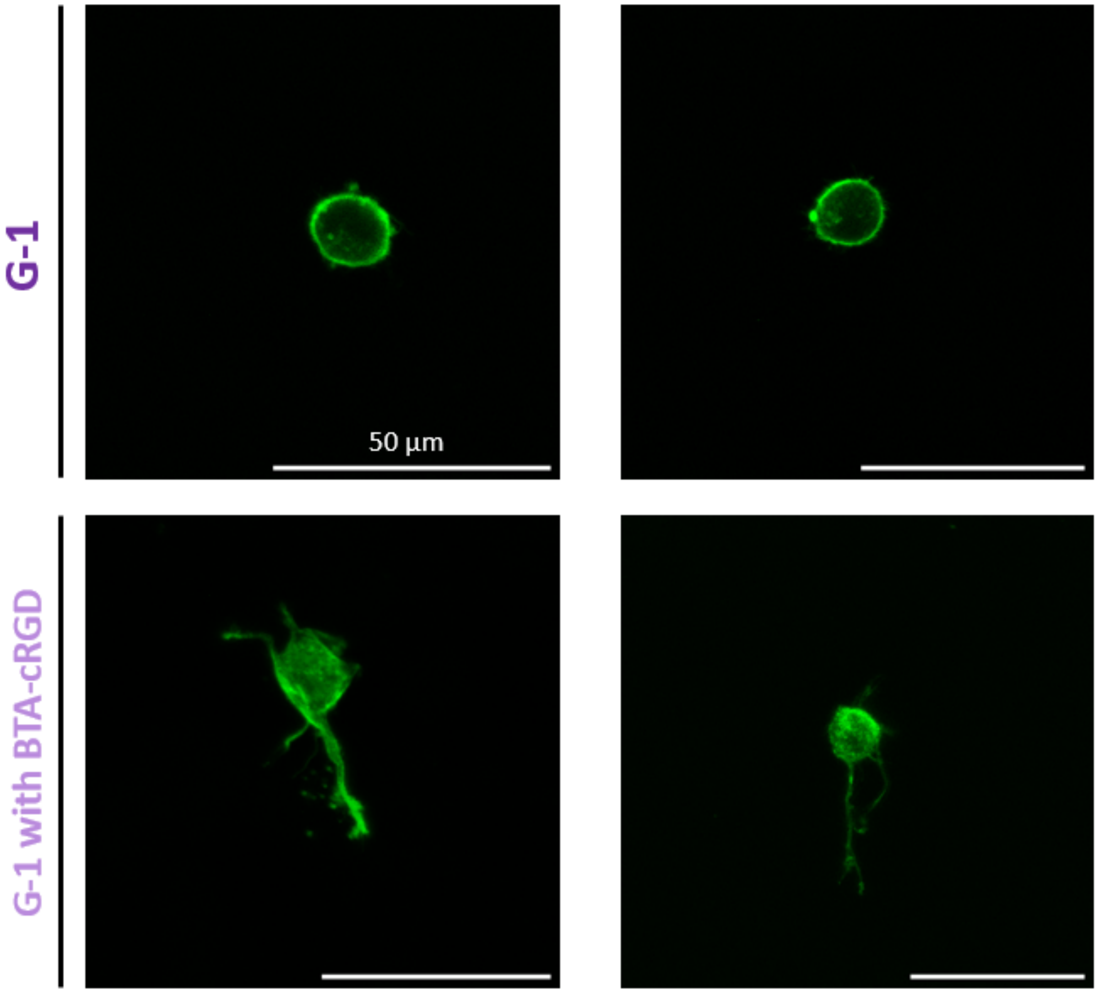

Figure S18: Additional cell images showing cells encapsulated in 3D inside hybrid supramolecular hydrogels after 1 day of culture. Green is F-actin, scale bar is 50 µm.

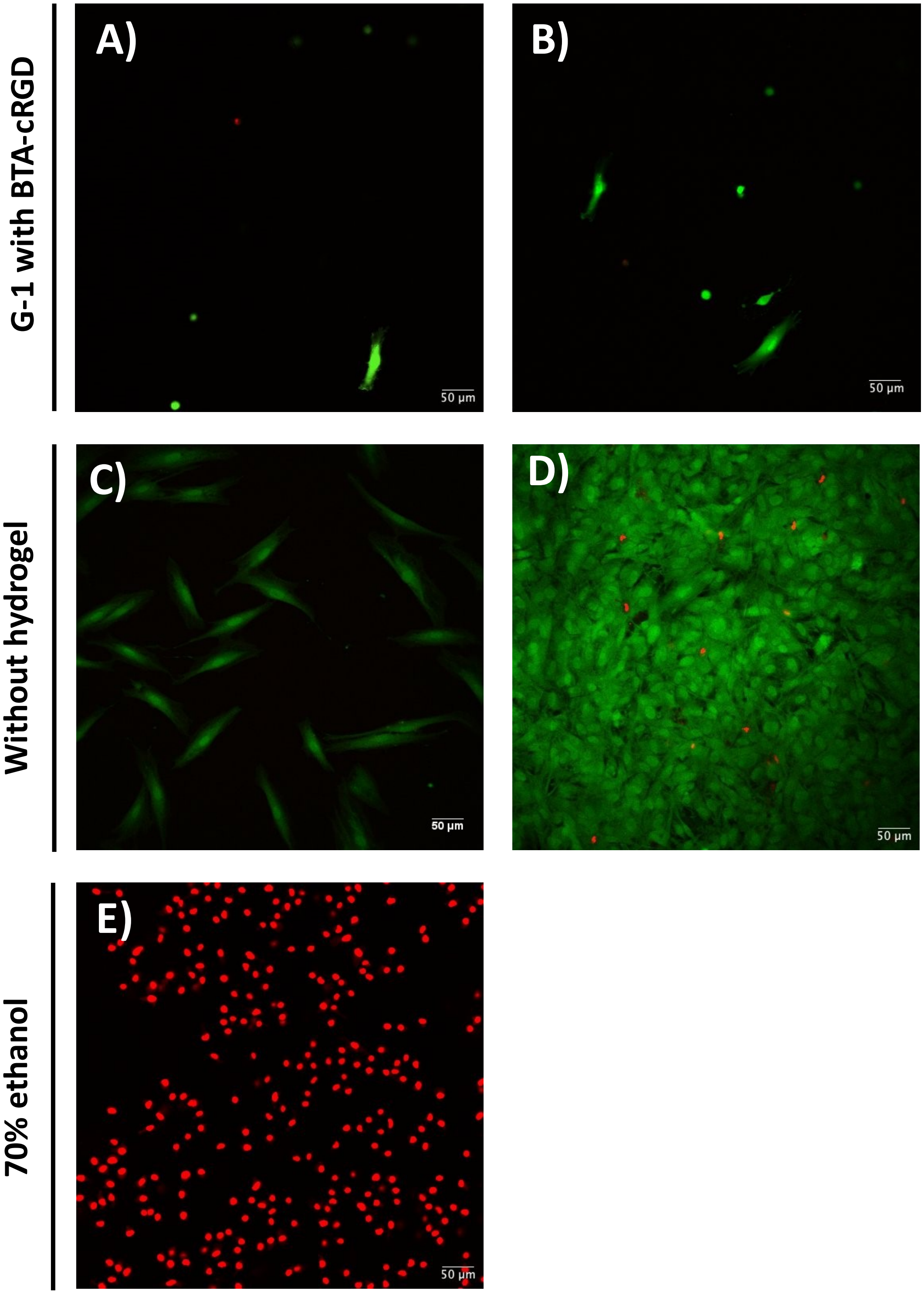

Figure S19: Additional cell images of live/dead cell staining (green=live, red=dead) after 24 h of culture. (**A, B**) Cells encapsulated in hydrogel **G-1** containing **BTA-cRGD** with 4000 cells/well. (**C)** Positive control of cells without hydrogel with 1000 cells/well. (**D**) Positive control of cells without hydrogel with 4000 cells/well. (**E**) Negative control of cells in 70% ethanol without hydrogel with 4000 cells/well. Scale bar = 50 µm.

References

(1) Albertazzi, L.; Van Der Zwaag, D.; Leenders, C. M. A.; Fitzner, R.; Van Der Hofstad, R. W.; Meijer, E. W. Probing Exchange Pathways in One-Dimensional Aggregates with Super-Resolution Microscopy. *Science* **2014**, *344* (6183), 491–495. https://doi.org/10.1126/SCIENCE.1250945.

(2) Rijns, L.; Peeters, J. W.; Hendrikse, S. I. S.; Vleugels, M. E. J.; Lou, X.; Janssen, H. M.; Meijer, E. W.; Dankers, P. Y. W. Importance of Molecular and Bulk Dynamics in Supramolecular Hydrogels in Dictating Cellular Spreading. *Chemistry of Materials* **2023**, *35* (19), 8203–8217. https://doi.org/10.1021/ACS.CHEMMATER.3C01676.
